# Synthesis of a Spirocyclic Oxetane-Fused Benzimidazole

**DOI:** 10.3390/molecules200813864

**Published:** 2015-07-30

**Authors:** Michael Gurry, Patrick McArdle, Fawaz Aldabbagh

**Affiliations:** School of Chemistry, National University of Ireland Galway, University Road, Galway SW4 NUI, Ireland; E-Mails: m.gurry1@nuigalway.ie (M.G.); patrick.mcardle@nuigalway.ie (P.M.)

**Keywords:** annulation, cyclization, diazole, heterocycle, 4-membered rings

## Abstract

A new synthesis of 2-oxa-7-azaspiro[3.5]nonane is described. Spirocyclic oxetanes, including 2-oxa-6-azaspiro[3.3]heptane were converted into *o*-cycloalkylaminoacetanilides for oxidative cyclizations using Oxone^®^ in formic acid. The expanded spirocyclic oxetane successfully gave the [1,2-*a*] ring-fused benzimidazole. X-ray crystal structure of the resultant new tetracyclic system, 1ʹ,2ʹ-dihydro-4ʹ*H*-spiro[oxetane-3,3ʹ-pyrido[1,2-*a*]benzimidazole] and the azetidine ring-opened adduct, *N*-(2-acetamido-4-bromophenyl)-*N*-{[3-(chloromethyl)oxetan-3-yl]methyl}acetamide are disclosed.

## 1. Introduction

**Figure 1:**
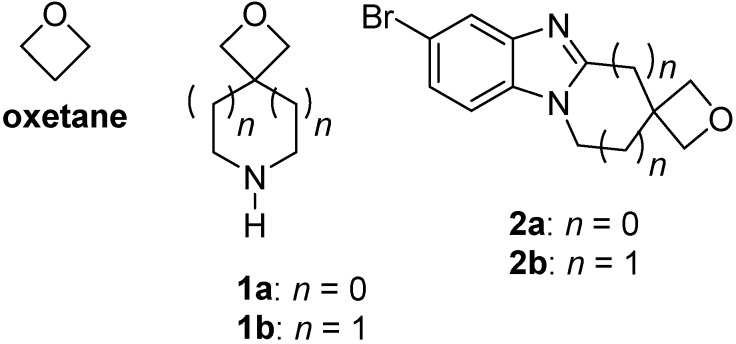
Rationale design of synthetic targets.

Oxetane is considered as a polar equivalent of the *gem*-dimethyl group (Figure 1) [1,2,3]. Spirocyclic oxetanes such as 2-oxa-6-azaspiro[3.3]heptane (**1a**) and 2-oxa-7-azaspiro[3.5]nonane (**1b**) were proposed as valuable structural alternatives to ubiquitous morpholine in medicinal chemistry [4]. A drug discovery project within our group demanded a substituent that enabled higher binding affinities to the NAD(P)H:quinone oxidoreductase 1 (NQO1) active site [5]. NQO1 is an enzyme over-expressed in cancer cell lines. Our attention turned to oxetane due to its heralded metabolic robustness in comparison to carbonyl alternatives, while offering the hydrogen bonding capacity necessary for efficient binding to the His194 residue of NQO1 enabling more efficient reduction of benzimidazolequinone and imidazobenzimidazolequinone substrates. The following article describes the first fusion of the spirocyclic oxetane motif onto heterocycles in attempts to prepare [1,2-*a*] alicyclic ring-fused benzimidazoles **2a** and **2b**.

## 2. Results and Discussion

### 2.1. Synthesis of Spirocyclic Oxetanes

**Scheme 1:**
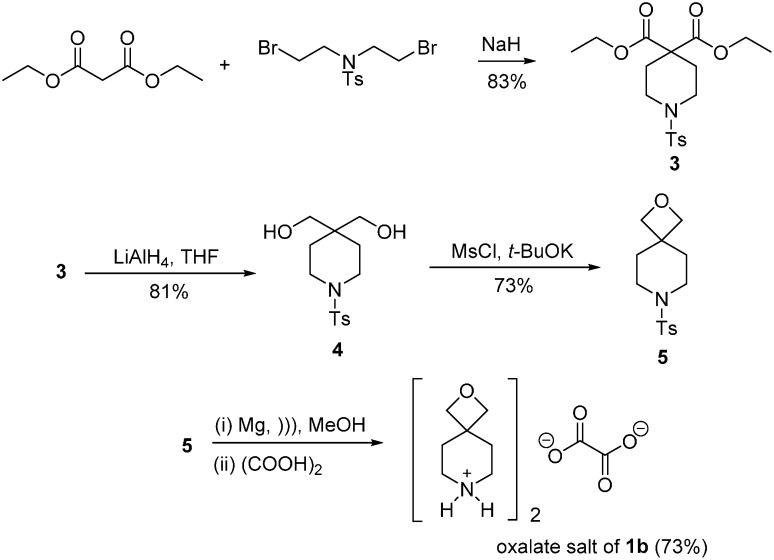
Synthesis of the oxalate salt of 2-oxa-7-azaspiro[3.5]nonane (**1b**).

The synthesis of the oxalate salt of 2-oxa-6-azaspiro[3.3]heptane **1a** starting from the condensation of 3-bromo-2,2-bis(bromomethyl)propan-1-ol with *p*-tosylamide has been described [4]. The expanded analogue **1b** is commercially available but expensive, and a new synthetic pathway is now reported (Scheme 1). *N*-tosyl-piperidine-4,4-diethyl ester **3** was prepared using the reaction of diethyl malonate with *N*-tosylbis(2-bromoethyl)amine, and similar transformations are available in patents [6,7]. Lithium aluminum hydride reduction provides diol **4**, which is subjected to a one-pot mesylation and ring closure to give the previously unreported oxetane **5**. The tosyl group was removed and the oxalate salt of 2-oxa-7-azaspiro[3.5]nonane **1b** formed. An alternative synthesis appears in a patent via the ring-closure of 2,2′-(oxetane-3,3-diyl)bis(ethan-1-ol) to give **1b** [8]. The latter multi-step synthesis begins with a Wittig reaction on 3-oxetanone, and represents a longer more costly preparation of **1b**.

### 2.2. Synthesis of o-Cycloalkylaminoacetanilides

**Scheme 2:**
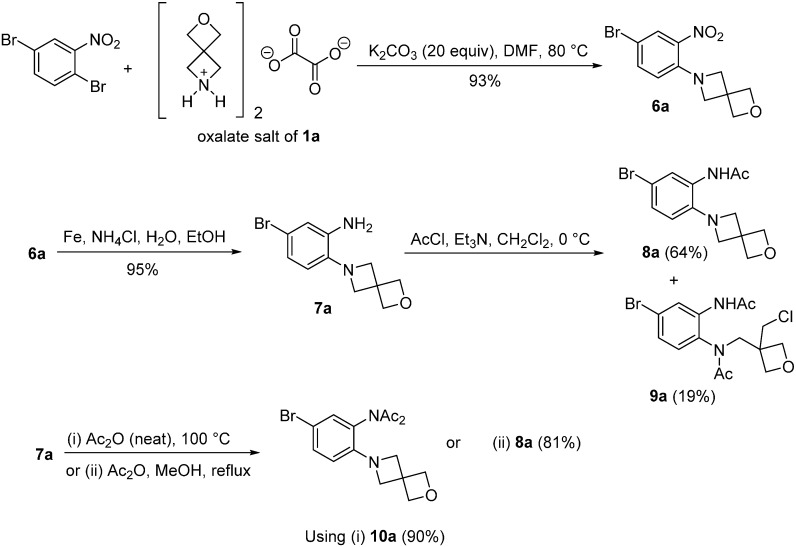
Synthesis of *N*-[5-bromo-2-(2-oxa-6-azaspiro[3.3]heptan-6-yl)phenyl]acetamide (**8a**).

**Scheme 3:**
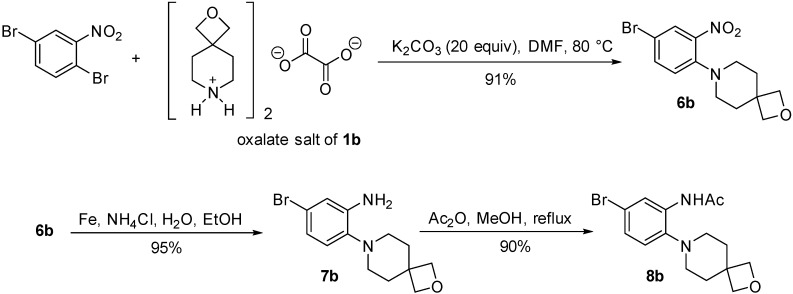
Synthesis of *N*-[5-bromo-2-(2-oxa-7-azaspiro[3.5]nonan-7-yl)phenyl]acetamide (**8b**).

**Figure 2:**
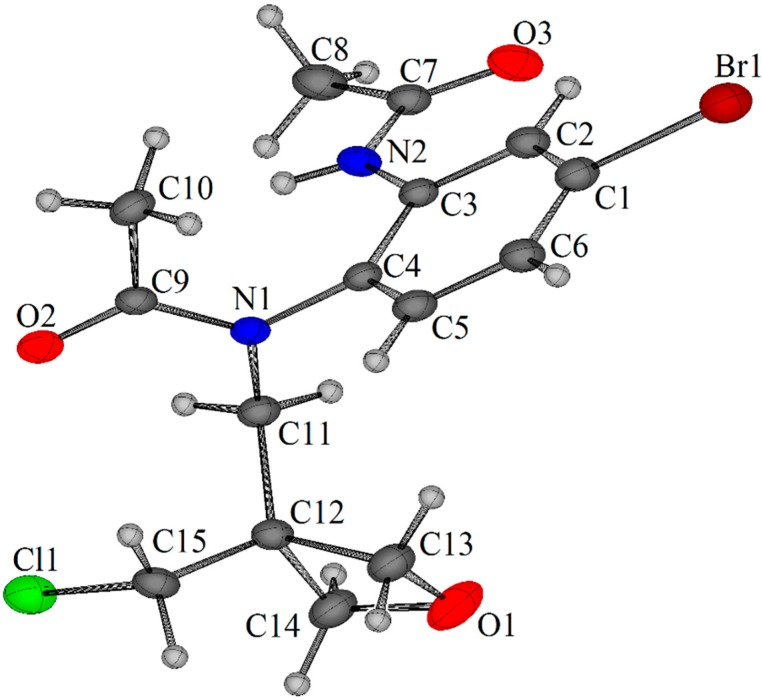
X-ray crystal structure of *N*-(2-acetamido-4-bromophenyl)-*N*-{[3-(chloromethyl)oxetan-3-yl]methyl}acetamide (**9a**) [10].

Nucleophilic aromatic substitution onto 1,4-dibromo-2-nitrobenzene using the oxalate salts of spirocyclic oxetanes **1a** and **1b** was possible in yields of >90% when using a large excess of potassium carbonate in DMF (Scheme 2 and Scheme 3). Reduction of the nitro group of **6a** and **6b** using iron and aqueous ammonium chloride yielded the corresponding anilines **7a** and **7b** in 95% yield. The acetylation was optimized with aniline **7a**, where the initial attempt using acetyl chloride gave the desired acetamide **8a** in 64% yield together with adduct **9a** of ring-opening of the azetidine in 19% yield (Scheme 2). By-product **9a** was confirmed by X-ray crystallography (Figure 2). As pointed out by the Reviewer this may have occurred using the chloride ion liberated from the first acetylation. The ring-opening of azetidines by the nucleophilic attack of halide ions on azetidinium ions has been previously described [9]. Acetylations using acetic anhydride were in contrast regioselective giving diacetylated adduct **10a** in 90% yield with neat acetic anhydride, while acetylation with acetic anhydride in methanol gave the desired product **8a** in 81% yield. The latter conditions gave the analogous acetamide of 2-oxa-7-azaspiro[3.5]nonane **8b** in 90% yield (Scheme 3).

### 2.3. Fusion of the Spirocyclic Oxetanes

**Scheme 4:**
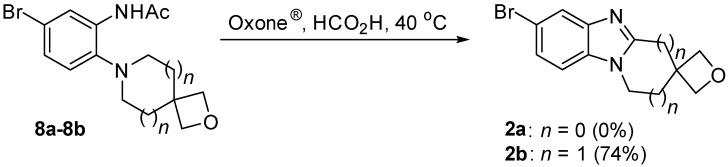
Oxidative cyclizations to give spirocyclic oxetane fused benzimidazoles.

**Figure 3:**
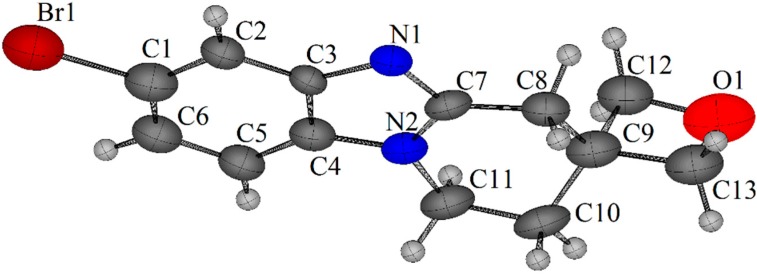
X-ray crystal structure of 7ʹ-bromo-1ʹ,2ʹ-dihydro-4ʹ*H*-spiro[oxetane-3,3ʹ-pyrido [1,2-*a*]benzimidazole] (**2b**) [10].

In order to fuse the spirocyclic oxetane of **8a** and **8b**, the well-established oxidative cyclization reaction of acetamide onto the neighbouring cyclic amine was used [11,12,13]. Oxone^®^ in formic acid is as an expedient means of converting *o*-cycloalkylaminoacetanilides into [1,2-*a*] alicyclic ring-fused benzimidazoles and double annulated imidazobenzimidazoles in high yields [13]. Using the latter conditions (Scheme 4), acetanilide **8a** gave a mixture of products indicating degradation of the spirocyclic oxetane and an absence of the desired benzimidazole **2a**. In contrast the 2-oxa-7-azaspiro[3.5]nonane fused benzimidazole **2b** was isolated in 74% yield at the end of the reaction of **8b** without the requirement for chromatography by simple organic extraction from the basified aqueous mixture. The structure of the novel tetracyclic system **2b** was confirmed by X-ray crystallography (Figure 3).

## 3. Experimental Section

### 3.1. General

All chemicals were obtained from commercial sources and used without further purification. Thin layer chromatography (TLC) was performed on TLC silica gel 60 F254 plates. Dry vacuum column chromatography was carried out on silica gel (Apollo Scientific ZEOprep 60/15–35 microns). Melting points were measured on a Stuart Scientific melting point apparatus SMP1. Infrared spectra were recorded using a Perkin-Elmer Spec 1 with ATR attached. ^1^H-NMR spectra were recorded using a Joel ECX 400 MHz instrument equipped with a DEC AXP 300 computer workstation. The chemical shifts were recorded in ppm relative to tetramethylsilane. ^13^C-NMR data were collected at 100 MHz with complete proton decoupling. High resolution mass spectra (HRMS) were carried out using ESI time-of-flight mass spectrometer (TOFMS). The precision of all accurate mass measurements were better than 5 ppm. NMR spectra of all compounds are available in the Supplementary Materials document accompanying this article.

### 3.2. Experimental Procedures

#### 3.2.1. Synthesis of Spirocyclic Oxetanes

*Bis*(2-oxa-6-azaspiro[3.3]heptan-6-ium) ethanedioate (oxalate salt of **1a**) was prepared according to the literature [4].

*Diethyl 1-(4-methylbenzene-1-sulfonyl)piperidine-4,4-dicarboxylate* (**3**). Diethyl malonate (0.500 g, 3.1 mmol) and NaH (0.165 g, 6.9 mmol) in DMF (50 mL) were heated at 80 °C for 30 min. *N*,*N*-bis(2-bromoethyl)-4-methyl-1-sulfonamide [14] (1.320 g, 3.4 mmol) was added and heating at 80 °C continued for 12 h. The cooled mixture was evaporated, EtOAc (50 mL) added, and washed with aqueous LiCl (5%) solution (3 × 50 mL). The organic extracts were dried (Na_2_SO_4_) and evaporated to dryness, and the residue purified by column chromatography using gradient elution of petroleum ether/EtOAc to yield the *title compound* (0.987 g, 83%) as a white solid; *R*_f_ 0.32 (1:4 EtOAc/Pet); mp 90–92 °C; ν_max_ (neat, cm^−1^) 2987, 2851, 1742 (C=O), 1598, 1467, 1444, 1367, 1350 (S=O), 1327, 1305, 1261, 1247, 1192, 1162 (S=O), 1092, 1052, 1072, 1015; δ_H_ (400 MHz, CDCl_3_) 1.16 (t, *J* = 7.3 Hz, 6H, CH_3_), 2.17 (t, *J* = 5.5 Hz, 4H), 2.41 (s, 3H), 3.03 (t, *J* = 5.5 Hz, 4H), 4.13 (q, *J* = 7.3 Hz, 4H, OCH_2_), 7.29 (d, *J* = 8.0 Hz, 2H), 7.59 (d, *J* = 8.0 Hz, 2H); δ_C_ (100 MHz, CDCl_3_) 14.0, 21.6 (CH_3_), 30.2, 43.2 (CH_2_), 52.4 (C), 61.8 (OCH_2_), 127.7, 129.8 (CH), 133.1, 143.7 (C), 170.4 (C=O); HRMS (ESI) *m*/*z* [M + H]^+^, C_18_H_26_NO_6_S calcd. 384.1481, observed 384.1483.

*[1-(4-Methylbenzene-1-sulfonyl)]piperidine-4,4-diyl]dimethanol* (**4**). LiAlH_4_ (5.2 mL of a 1 M solution in THF, 5.2 mmol) was added over 5 min to a solution of diethyl ester **3** (1.000 g, 2.6 mmol) in THF (20 mL), and the solution stirred for 16 h at room temperature. The reaction was quenched with ethanol (3 mL), a saturated sodium potassium tartrate solution (40 mL), and stirred vigorously for 1 h. The resulting biphasic mixture was extracted with EtOAc (2 × 50 mL), washed with brine (2 × 30 mL) and dried (Na_2_SO_4_). The solution was evaporated to dryness and the residue purified by column chromatography using gradient elution of petroleum ether/EtOAc to yield the *title compound* (0.628 g, 81%) as a white solid; *R*_f_ 0.45 (EtOAc); mp 110–114 °C; ν_max_ (neat, cm^−1^) 3524 (OH), 3257, 2919, 2869, 1725, 1597, 1340, 1320 (S=O), 1248, 1184, 1156 (S=O), 1132, 1090, 1053, 1012; δ_H_ (400 MHz, CDCl_3_) 1.57 (t, *J* = 5.7 Hz, 4H), 2.43 (s, 3H), 2.56 (t, *J* = 4.9 Hz, 2H, OH, disappears with D_2_O), 2.97 (t, *J* = 5.7 Hz, 4H), 3.49 (d, *J* = 4.9 Hz, 4H, C*H*_2_OH), 7.31 (d, *J* = 7.8 Hz, 2H), 7.61 (d, *J* = 7.8 Hz, 2H); δ_C_ (100 MHz, CDCl_3_) 21.6 (CH_3_), 28.4 (CH_2_), 36.6 (C), 42.1 (CH_2_), 68.7 (OCH_2_), 127.8, 129.8 (CH), 132.9, 143.8 (C); HRMS (ESI) *m*/*z* [M + H]^+^, C_14_H_22_NO_4_S calcd. 300.1270, observed 300.1269.

*7-(4-Methylbenzene-1-sulfonyl)-2-oxa-7-azaspiro[*3.5]*nonane* (**5**). Methanesulfonyl chloride (0.4 mL, 5.2 mmol) in THF (5 mL) was added to diol **4** (1.700 g, 5.7 mmol) in THF (50 mL) via syringe pump at a rate of 1.7 mL/h, while potassium *tert*-butoxide (0.636 g, 5.7 mmol) was added in portions (one third per h). The mixture was stirred at room temperature for 1 h with further potassium *tert*-butoxide (1.900 g, 17.0 mmol) added, and stirred for 2 h. The mixture was evaporated, EtOAc (50 mL) added, washed with water (2 × 20 mL), and dried (Na_2_SO_4_). The solution was evaporated to dryness and the residue purified by column chromatography using gradient elution of petroleum ether/EtOAc to yield the title compound (1.160 g, 73%) as a white solid; *R*_f_ 0.44 (1:1 EtOAc/Pet); mp 168–172 °C; ν_max_ (neat, cm^−1^) 2928, 2868, 1597, 1597, 1345, 1331 (S=O), 1241, 1163 (S=O), 1140, 1089; δ_H_ (400 MHz, CDCl_3_) 1.93 (bs, 4H), 2.42 (s, 3H), 2.90 (bs, 4H), 4.29 (s, 4H, OCH_2_), 7.30 (d, *J* = 7.3 Hz, 2H), 7.60 (d, *J* = 7.3 Hz, 2H); δ_C_ (100 MHz, CDCl_3_), 21.7 (CH_3_) 34.1 (CH_2_), 38.1 (C), 43.3 (CH_2_), 81.1 (OCH_2_), 127.7, 129.8 (CH), 133.1, 143.8 (C); HRMS (ESI) *m*/*z* [M + H]^+^, C_14_H_20_NO_3_S calcd. 282.1164, observed 282.1163.

*Bis(2-oxa-7-azaspiro*[3.5]*nonan-7-ium) ethanedioate* (oxalate salt of **1b**). 2-Oxa-7azaspiro[3.5]nonane **5** (1.300 g, 4.6 mmol) and Mg turnings (0.780 g, 32.3 mmol) in MeOH (50 mL) were sonicated for 1 h. The mixture was evaporated to give a viscous grey residue to which Et_2_O (50 mL) and Na_2_SO_4_·10H_2_O (2.000 g) were added. After 30 min of stirring, the mixture was filtered, and anhydrous oxalic acid (0.210 g, 2.3 mmol) added to the filtrate to form a precipitate. The precipitate was collected and dried under vacuum to yield the *title compound* (0.580 g, 73%) as a white solid; mp 148–152 °C; ν_max_ (neat, cm^−1^) 3389 (NH), 3124, 2931, 2866, 1713, 1610 (C=O), 1475, 1444, 1397, 1171; δ_H_ (400 MHz, D_2_O) 1.94 (t, *J* = 5.7 Hz, 4H), 2.98 (t, *J* = 5.7 Hz, 4H), 4.38 (s, 4H, OCH_2_); δ_C_ (100 MHz, D_2_O) 30.2 (CH_2_), 36.6 (C), 41.1 (CH_2_), 81.1 (OCH_2_), 165.7 (C=O).

#### 3.2.2. Synthesis of Acetanilide Cyclization Precursors

##### General Procedure for the Synthesis of Nitrobenzenes **6a** and **6b**

1,4-Dibromo-2-nitrobenzene (1.000 g, 3.6 mmol), K_2_CO_3_ (9.820 g, 71.2 mmol) and spirocyclic oxetane **1a**–**1b** oxalate salts (3.6 mmol) were heated in DMF (50 mL) at 80 °C for 4 h. The cooled mixture was evaporated, EtOAc (50 mL) added, and washed with aqueous LiCl (5%) solution (3 × 20 mL). The organic extracts were dried (Na_2_SO_4_), evaporated to dryness, and purified by column chromatography using gradient elution of petroleum ether/EtOAc to yield:

*6-(4-Bromo-2-nitrophenyl)-2-oxa-6-azaspiro[*3.3]*heptane* (**6a**). (0.987 g, 93%) as an orange solid; *R*_f_ 0.49 (1:1 EtOAc/Pet); mp 148–150 °C; ν_max_ (neat, cm^−1^) 2955, 2861, 1672, 1602, 1558 (NO_2_), 1502, 1484, 1458, 1373, 1362, 1348 (NO_2_), 1276, 1246, 1203, 1123, 1070; δ_H_ (400 MHz, CDCl_3_) 4.11 (s, 4H, NCH_2_), 4.81 (s, 4H, OCH_2_), 6.47 (d, *J* = 9.2 Hz, 1H, 6-H), 7.45 (dd, *J* = 9.2, 2.3 Hz, 1H, 5-H), 7.93 (d, *J* = 2.3 Hz, 1H, 3-H); δ_C_ (100 MHz, CDCl_3_) 38.3 (C), 62.8 (NCH_2_), 80.9 (OCH_2_), 108.3 (C), 117.4 (6-CH), 128.8 (3-CH), 136.2 (C), 136.5 (5-CH), 143.8 (C); HRMS (ESI) *m*/*z* [M + H]^+^, C_11_H_12_N_2_O_3_^79^Br calcd. 299.0031, observed 299.0038.

*7-(4-Bromo-2-nitrophenyl)-2-oxa-7-azaspiro[*3.5]*nonane* (**6b**). (1.061 g, 91%) as an orange oil; *R*_f_ 0.58 (1:4 EtOAc/Pet); ν_max_ (neat, cm^−1^) 2931, 2861, 1599, 1519 (NO_2_), 1485, 1462, 1384, 1331 (NO_2_), 1271, 1231, 1168, 1131, 1091; δ_H_ (400 MHz, CDCl_3_) 1.99–2.02 (m, 4H), 2.90–2.93 (m, 4H, NCH_2_), 4.46 (s, 4H, OCH_2_), 6.97 (d, *J* = 8.7 Hz, 1H, 6-H), 7.53 (dd, *J* = 8.7, 2.3 Hz, 1H, 5-H), 7.89 (d, *J* = 2.3 Hz, 1H, 3-H); δ_C_ (100 MHz, CDCl_3_) 34.9 (CH_2_) 38.3 (C), 49.2 (NCH_2_), 81.6 (OCH_2_), 113.3 (C), 122.8 (6-CH), 128.7 (3-CH), 136.4 (5-CH), 143.3, 145.5 (C); HRMS (ESI) *m*/*z* [M + H]^+^, C_13_H_16_N_2_O_3_^79^Br calcd. 327.0344, observed 327.0355.

##### General Procedure for the Synthesis of Anilines **7a** and **7b**

Nitrobenzene **6a** or **6b** (2.00 mmol), Fe powder (0.370 g, 6.6 mmol), NH_4_Cl (59 mg, 1.0 mmol) and water (1.5 mL) in EtOH (5 mL) were heated at 80 °C for 5 h. EtOAc (50 mL) was added to the cooled mixture, which was filtered, washed with water (50 mL), and dried to yield:

*5-Bromo-2-(2-oxa-6-azaspiro[*3.3]*heptan-6-yl)aniline* (**7a**). (0.508 g, 95%) as a brown oil; ν_max_ (neat, cm^−1^) 3414, 3334, 2940, 2870, 1614, 1575, 1497, 1292, 1255, 1134, 1046; δ_H_ (400 MHz, CDCl_3_) 3.50 (bs, 2H, NH_2_), 3.91 (s, 4H, NCH_2_), 4.82 (s, 4H, OCH_2_), 6.41 (d, *J* = 8.4 Hz, 1H, 3-H), 6.75 (d, *J* = 2.0 Hz, 1H, 6-H), 6.82 (dd, *J* = 8.4, 2.0 Hz, 1H, 4-H); δ_C_ (100 MHz, CDCl_3_) 39.1 (C), 62.1 (NCH_2_), 81.2 (OCH_2_), 114.1 (C), 115.8 (3-CH), 118.4 (6-CH), 121.7 (4-CH), 137.8 (C), 139.1 (C); HRMS (ESI) *m*/*z* [M + H]^+^, C_11_H_14_N_2_O^79^Br calcd. 269.0289, observed 269.0290.

*5-Bromo-2-(2-oxa-7-azaspiro[*3.5]*nonan-7-yl)aniline* (**7b**). (0.564 g, 95%) as a brown solid; mp 160–162 °C; ν_max_ (neat, cm^−1^) 3400, 3317, 2931, 2865, 2811, 1618, 1577, 1493, 1459, 1231, 1210, 1133, 1122; δ_H_ (400 MHz, CDCl_3_) 1.98 (bs, 4H), 2.72 (bs, 4H, NCH_2_), 3.99 (bs, 2H, NH_2_), 4.47 (s, 4H, OCH_2_), 6.76–6.84 (m, 3H); δ_C_ (100 MHz, CDCl_3_) 35.8 (CH_2_), 38.5 (C), 48.7 (NCH_2_), 81.9 (OCH_2_), 117.6 (C & CH), 121.2, 121.4 (CH), 138.7, 143.1 (C); HRMS (ESI) *m*/*z* [M − H]^−^, C_13_H_16_N_2_O^79^Br calcd. 295.0446, observed 295.0448.

##### Procedures for the Synthesis of *N*-[5-Bromo-2-(2-oxa-6-azaspiro[3.3]heptan-6-yl)phenyl]acetamide (**8a**)

AcCl (0.2 mL, 2.5 mmol) was added over 5 min to aniline **7a** (0.420 g, 1.6 mmol) and Et_3_N (0.4 mL, 2.7 mmol) in CH_2_Cl_2_ (10 mL) at 0 °C, and stirred for 3 h at room temperature. The solution was evaporated, EtOAc (50 mL) added, and washed with water (50 mL). The organic extracts were dried (Na_2_SO_4_), evaporated, and purified by column chromatography using gradient elution of petroleum ether/EtOAc to yield:

*N-(2-acetylamido-4-bromophenyl)-N*-{[3-(chloromethyl)oxetan-3-yl]*methyl}acetamide* (**9a**). 0.114 g, 19%; brown solid; mp 160–164 °C; *R*_f_ 0.25 (EtOAc); ν_max_ (neat, cm^−1^) 3296, 3262, 2953, 2876, 1691 (C=O), 1657 (C=O), 1578, 1518, 1403, 1364, 1313, 1259, 1193, 1089; δ_H_ (400 MHz, CDCl_3_) 1.85 (s, 3H), 2.12 (s, 3H), 3.57 (d, *J* = 14.2 Hz, 1H), 3.67 (d, *J* = 11.2 Hz, 1H), 3.79 (d, *J* = 11.2 Hz, 1H), 4.12 (d, *J* = 7.1 Hz, 1H, C*H*HO), 4.42 (d, *J* = 14.2 Hz, 1H), 4.59–4.63 (m, 2H, OCH_2_), 4.68 (d, *J* = 7.1 Hz, 1H, CH*H*O), 7.09 (d, *J* = 8.5 Hz, 1H, 6-H), 7.32 (dd, *J* = 8.5, 2.1 Hz, 1H, 5-H), 8.43 (d, *J* = 2.1 Hz, 1H, 3-H), 9.18 (bs, 1H, NH); δ_C_ 22.3, 24.2 (CH_3_), 45.9 (C), 48.3, 53.2 (CH_2_), 76.8, 77.2 (OCH_2_), 122.8 (C), 127.0 (3-CH), 128.8 (2 × CH), 133.9, 137.2 (C), 169.3, 173.7 (C=O); HRMS (ESI) *m*/*z* [M + H]^+^, C_15_H_19_N_2_O_3_^35^Cl^79^Br calcd. 389.0268, observed 389.0271 and *N*-[5-bromo-2-(2-oxa-6-azaspiro[3.3]*heptan-6-yl)phenyl]acetamide* (**8a**). (0.312 g, 64%) as a white solid; *R*_f_ 0.18 (EtOAc); mp 185–189 °C; ν_max_ (neat, cm^−1^) 3218, 2929, 2864 1652 (C=O), 1591, 1569, 1520, 1485, 1408, 1368, 1317, 1279, 1254, 1132, 1060, 1011; δ_H_ (400 MHz, (CD_3_)_2_SO) 1.99 (s, 3H, CH_3_), 3.94 (s, 4H, NCH_2_), 4.65 (s, 4H, OCH_2_), 6.43 (d, *J* = 8.7 Hz, 1H, 3-H), 7.15 (dd, *J* = 8.7, 2.3 Hz, 1H, 4-H), 7.25 (d, *J* = 2.3 Hz, 1H, 6-H), 9.23 (s, 1H, NH); δ_C_ (100 MHz, (CD_3_)_2_SO) 23.7 (CH_3_), 39.0 (C), 62.4 (NCH_2_), 80.3 (OCH_2_), 109.3 (C), 115.9 (3-CH), 126.5 (C), 129.0 (4-CH), 130.4 (6-CH), 145.7 (C), 169.2 (C=O); HRMS (ESI) *m*/*z* [M − H]^−^, C_13_H_14_N_2_O_2_^79^Br calcd. 309.0239, observed 309.0249.

*N-Acetyl-N*-[5-bromo-2-(2-oxa-6-azaspiro[3.3]*heptan-6-yl)phenyl]acetamide* (**10a**). Aniline **7a** (0.850 g, 3.2 mmol) in Ac_2_O (30 mL) was heated at 100 °C for 2 h. The cooled solution was evaporated, ice-water (50 mL) added, and stirred for 3 h. The resultant precipitate was purified by column chromatography using gradient elution of petroleum ether/EtOAc to yield the *title compound* (1.020 g, 90%) as a white solid; *R*_f_ 0.34 (1:1 EtOAc/Pet); mp 152–156 °C; ν_max_ (neat, cm^−1^) 2932, 2861, 1707 (C=O), 1702 (C=O), 1591, 1493, 1479, 1458, 1408, 1366, 1334, 1300, 1284, 1225, 1156, 1126, 1073, 1022; δ_H_ (400 MHz, CDCl_3_) 2.29 (s, 6H, CH_3_), 3.96 (s, 4H, NCH_2_), 4.76 (s, 4H, OCH_2_), 6.41 (d, *J* = 8.7 Hz, 1H, 3-H), 7.05 (d, *J* = 2.3 Hz, 1H, 6-H), 7.34 (dd, *J* = 8.7, 2.3 Hz, 1H, 4-H); δ_C_ 26.5 (CH_3_), 39.0 (C), 62.2 (NCH_2_), 80.9 (OCH_2_), 111.1 (C), 115.9 (3-CH), 126.7 (C) , 132.7, 133.0 (CH), 146.4 (C), 174.0 (C=O); HRMS (ESI) *m*/*z* [M + H]^+^, C_15_H_18_N_2_O_3_^79^Br calcd. 353.0501, observed 353.0501.

*N*-[5-Bromo-2-(2-oxa-7-azaspiro[3.5]*nonan-7-yl)phenyl]acetamide* (**8b**). Ac_2_O (0.9 mL, 10.0 mmol) and aniline **7b** (0.594 g, 2.0 mmol) in methanol (20 mL) were heated at reflux for 1 h. The cooled mixture was evaporated, ice-water added (40 mL), and stirred for 3 h. The resultant precipitate was washed with water, and dried under vacuum to yield the *title compound* (0.611 g, 90%) as a white solid; mp 164–166 °C; ν_max_ (neat, cm^−1^) 3353, 2934, 2860, 2811, 1687 (C=O) 1578, 1507, 1445, 1410, 1372, 1224, 1132, 1109; δ_H_ (400 MHz, CDCl_3_) 2.02 (bs, 4H), 2.18 (s, 3H), 2.69 (bs, 4H, NCH_2_), 4.48 (s, 4H, OCH_2_), 6.92 (d, *J* = 8.2 Hz, 1H, 3-H), 7.12 (dd, *J* = 8.2, 1.8 Hz, 1H, 4-H), 8.36 (bs, 1H, NH), 8.53 (d, *J* = 1.8 Hz, 1H, 6-H); δ_C_ (100 MHz, CDCl_3_) 25.0 (CH_3_), 35.8 (CH_2_), 38.3 (C), 49.9 (NCH_2_), 81.6 (OCH_2_), 118.8 (C), 121.8 (3-CH), 122.3 (6-CH), 126.6 (4-CH), 134.6, 140.2 (C), 168.1 (C=O); HRMS (ESI) *m*/*z* [M + H]^+^, C_15_H_20_N_2_O_2_^79^Br calcd. 339.0708, observed 339.0707.

#### 3.2.3. Oxidative Cyclization to Give the Spirocyclic Oxetane Fused Benzimidazole

*7ʹ-Bromo-1ʹ,2ʹ-dihydro-4ʹH-spiro[oxetane-3,3ʹ-pyrido[1,2-a]benzimidazole]* (**2b**). Acetanilide **8b** (0.150 g, 0.44 mmol) and Oxone^®^ (0.820 g, 1.32 mmol) were stirred in formic acid (20 mL) at 40 °C for 6 h. The mixture was evaporated, water added (30 mL), neutralized with solid Na_2_CO_3,_ and extracted with CH_2_Cl_2_ (3 × 10 mL). The organic extracts were dried (Na_2_SO_4_), and evaporated dryness to yield the *title compound* (96 mg, 74%) as a white solid; mp 178–181 °C; ν_max_ (neat, cm^−1^) 2926, 2856, 1721, 1511, 1481, 1450, 1406, 1307, 1269, 1163, 1047; δ_H_ (400 MHz, CDCl_3_) 2.44 (t, *J* = 6.4 Hz, 2H, 2ʹ-CH_2_), 3.36 (s, 2H, 4ʹ-CH_2_), 4.09 (t, *J* = 6.4 Hz, 2H, 1ʹ-CH_2_), 4.54–4.59 (m, 4H, 2,4-CH_2_), 7.14 (d, *J* = 8.7 Hz, 1H, 9ʹ-H), 7.32 (dd, *J* = 8.7, 1.8 Hz, 1H, 8ʹ-H), 7.79 (d, *J* = 1.8 Hz, 1H, 6ʹ-H); δ_C_ (100 MHz, CDCl_3_) 30.6 (2ʹ-CH_2_), 34.9 (4ʹ-CH_2_), 37.9 (C), 39.0 (1'-CH_2_), 80.6 (2,4-CH_2_), 110.2 (9ʹ-CH), 115.6 (C), 122.1 (6ʹ-CH), 125.2 (8ʹ-CH), 133.3, 144.5, 150.8 (C); HRMS (ESI) *m*/*z* [M + H]^+^, C_13_H_14_N_2_O^79^Br calcd. 293.0289, observed 293.0287.

## 4. Conclusions

The new spirocyclic oxetane fused system, 1ʹ,2ʹ-dihydro-4ʹ*H*-spiro[oxetane-3,3ʹ-pyrido[1,2-*a*]benzimidazole] has been prepared through oxidative cyclization of the *o*-cycloalkylaminoacetanilide, where 2-oxa-7-azaspiro[3.5]nonane is the cycloamino substituent. Future work will focus on preparing quinone analogues for anti-cancer studies, as well as investigating conditions for the synthesis of the more strained tetracycle **2a**. The acetanilide containing 2-oxa-6-azaspiro[3.3]heptane system proved to be unstable under the presented oxidative cyclization conditions giving an intractable mixture. Research is on-going in the group to establish milder conditions for oxidative cyclizations of *o*-cyclic amine substituted anilines [15] and acetamides to give ring-fused benzimidazoles.

## Supplementary Materials

Supplementary materials can be accessed at: http:www.mdpi.com/1420-3049/20/08/13864/s1.
