# Supplementary material for: Synthesis of a Spirocyclic Oxetane-Fused Benzimidazole
Source: Molecules. 2015 Jul 30;20(8):13864–74. doi: 10.3390/molecules200813864 (PMC6332447; doi:10.3390/molecules200813864)

Supplementary

^{1}H-NMR (400 MHz) and ^{13}C-NMR (100 MHz) Spectra

Diethyl 1-(4-methylbenzene-1-sulfonyl)piperidine-4,4-dicarboxylate (**3**) **S2**

[1-(4-Methylbenzene-1-sulfonyl)]piperidine-4,4-diyl]dimethanol (**4**) **S4**

7-(4-Methylbenzene-1-sulfonyl)-2-oxa-7-azaspiro[3.5]nonane (**5**) **S6**

Bis(2-oxa-7-azaspiro[3.5]nonan-7-ium) ethanedioate (oxalate salt of **1b**) **S8**

6-(4-Bromo-2-nitrophenyl)-2-oxa-6-azaspiro[3.3]heptane (**6a**) **S10**

7-(4-Bromo-2-nitrophenyl)-2-oxa-7-azaspiro[3.5]nonane (**6b**) **S12**

5-Bromo-2-(2-oxa-6-azaspiro[3.3]heptan-6-yl)aniline (**7a**) **S14**

5-Bromo-2-(2-oxa-7-azaspiro[3.5]nonan-7-yl)aniline (**7b**) **S16**

N-(2-Acetylamido-4-bromophenyl)-*N*-{[3-(chloromethyl)oxetan-3-yl]methyl}acetamide (**9a**) **S18**

N-[5-Bromo-2-(2-oxa-6-azaspiro[3.3]heptan-6-yl)phenyl]acetamide (**8a**) **S20**

N-Acetyl-*N*-[5-bromo-2-(2-oxa-6-azaspiro[3.3]heptan-6-yl)phenyl]acetamide (**10a**) **S22**

N-[5-Bromo-2-(2-oxa-7-azaspiro[3.5]nonan-7-yl)phenyl]acetamide (**8b**) **S24**

7′-Bromo-1′,2′-dihydro-4′*H*-spiro[oxetane-3,3′-pyrido[1,2-*a*]benzimidazole] (**2b**) **S26**

^{1}H-NMR (400 MHz) of Diethyl 1-(4-methylbenzene-1-sulfonyl)piperidine-4,4-dicarboxylate (**3**) in $CDCl_3$

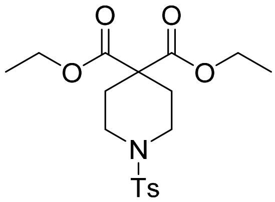

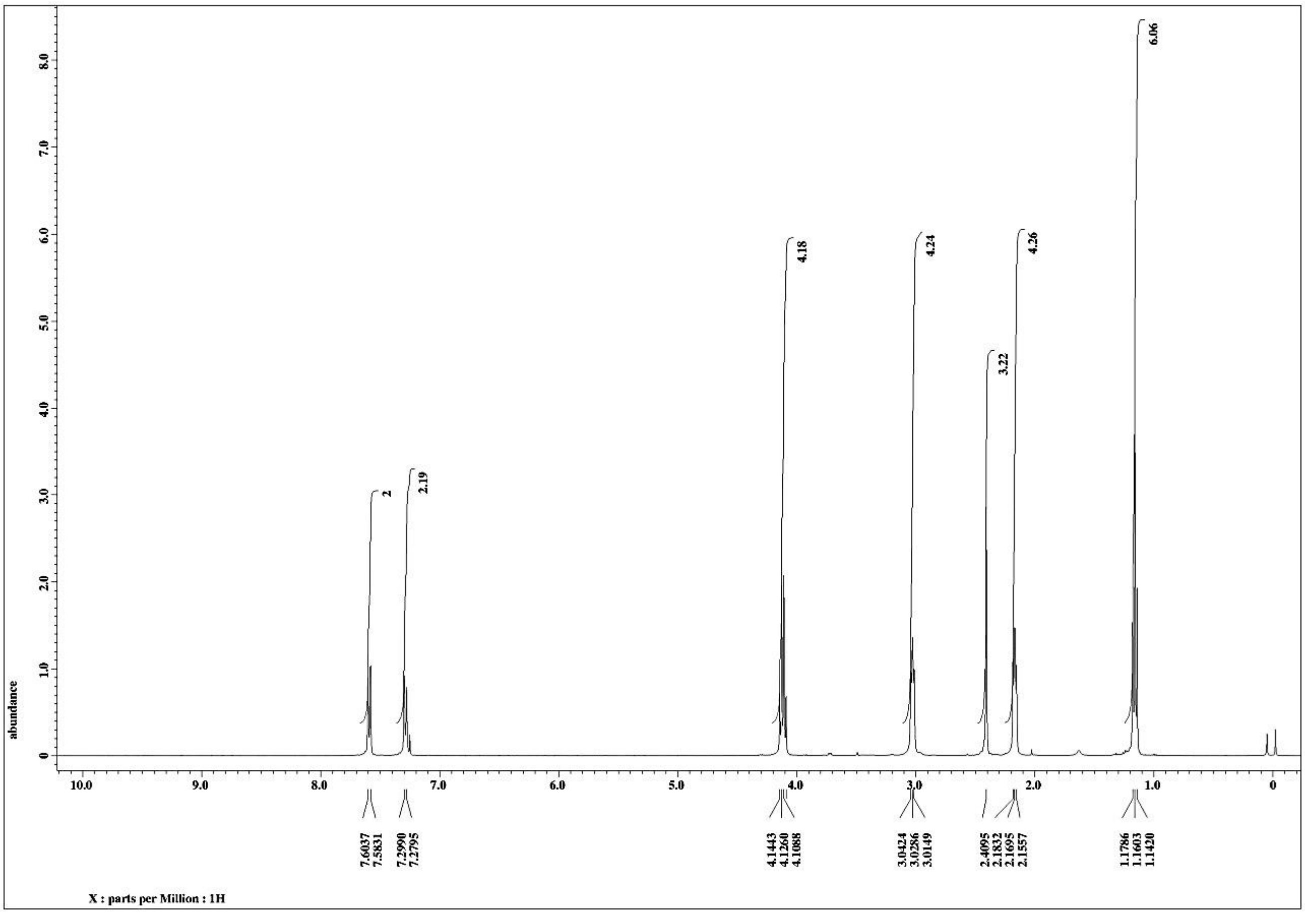

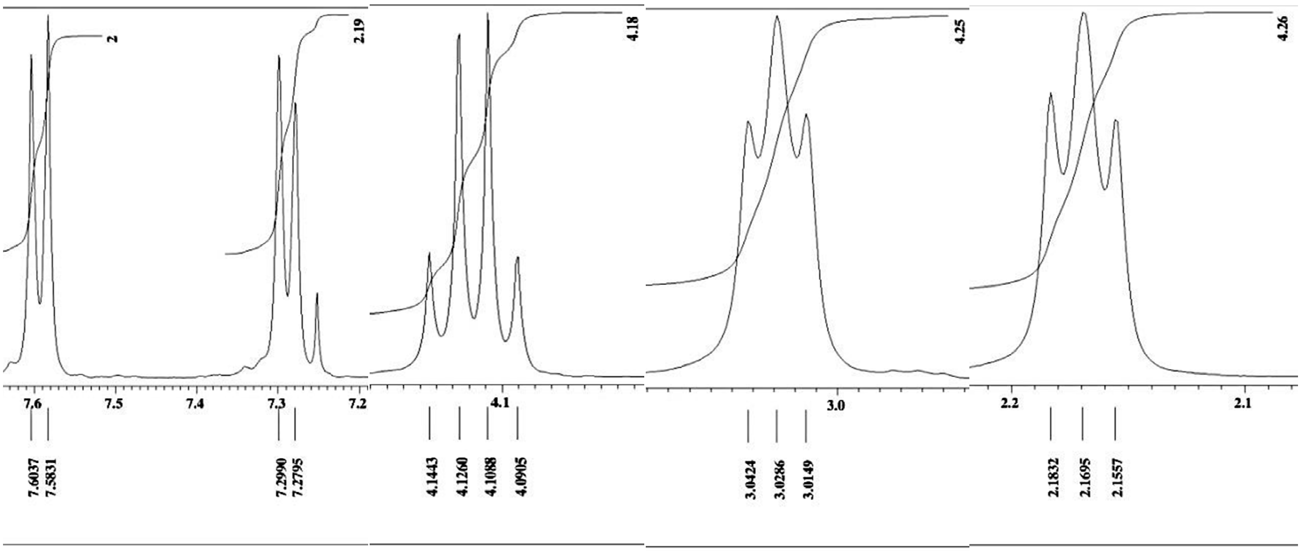

^{13}C-NMR (100 MHz) of Diethyl 1-(4-methylbenzene-1-sulfonyl)piperidine-4,4-dicarboxylate (**3**) in $CDCl_3$

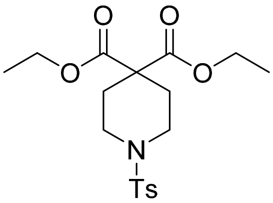

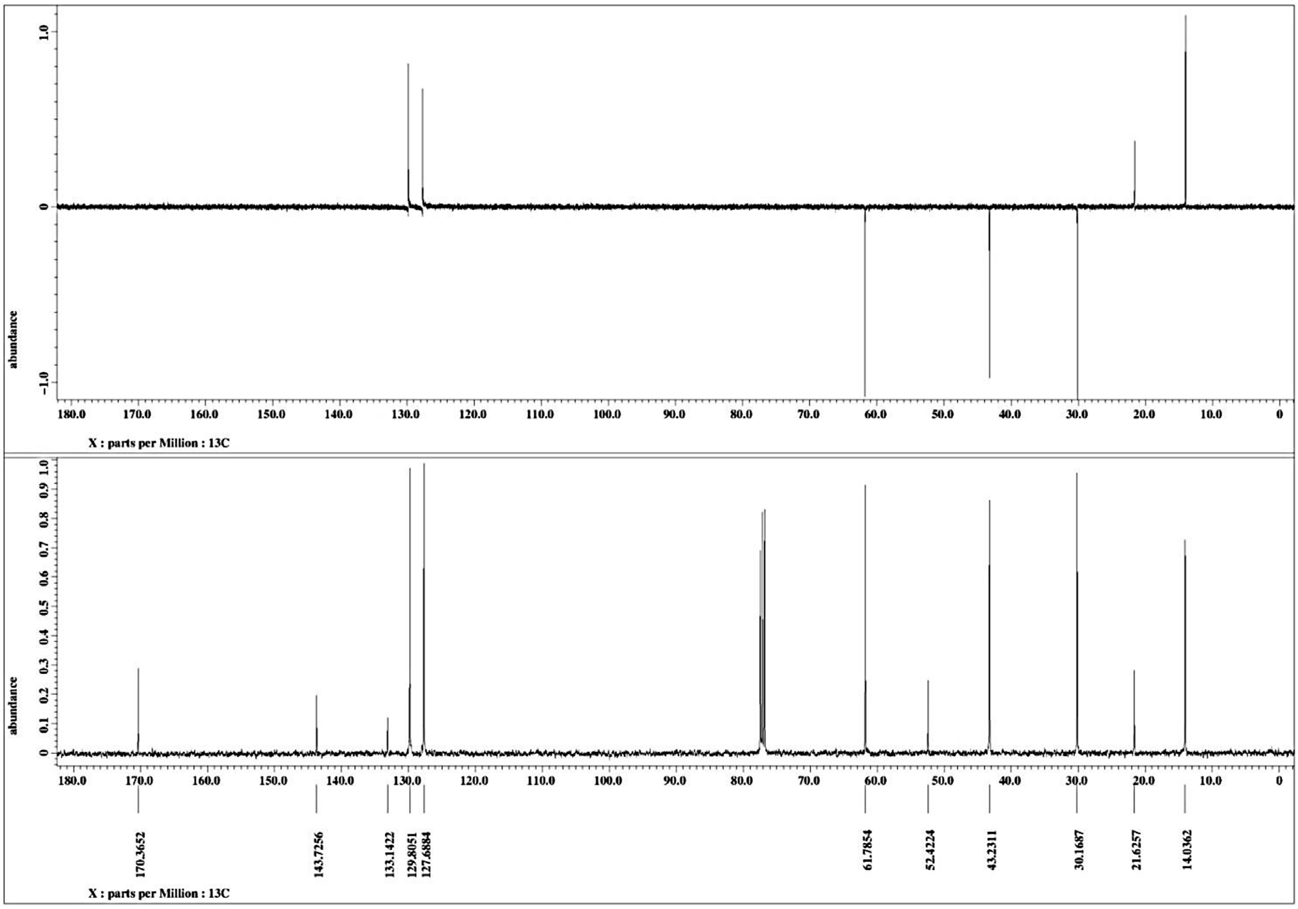

^{1}H-NMR (400 MHz) of [1-(4-Methylbenzene-1-sulfonyl)]piperidine-4,4-diyl]dimethanol (**4**) in $CDCl_3$

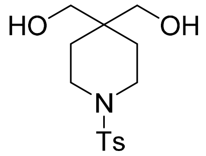

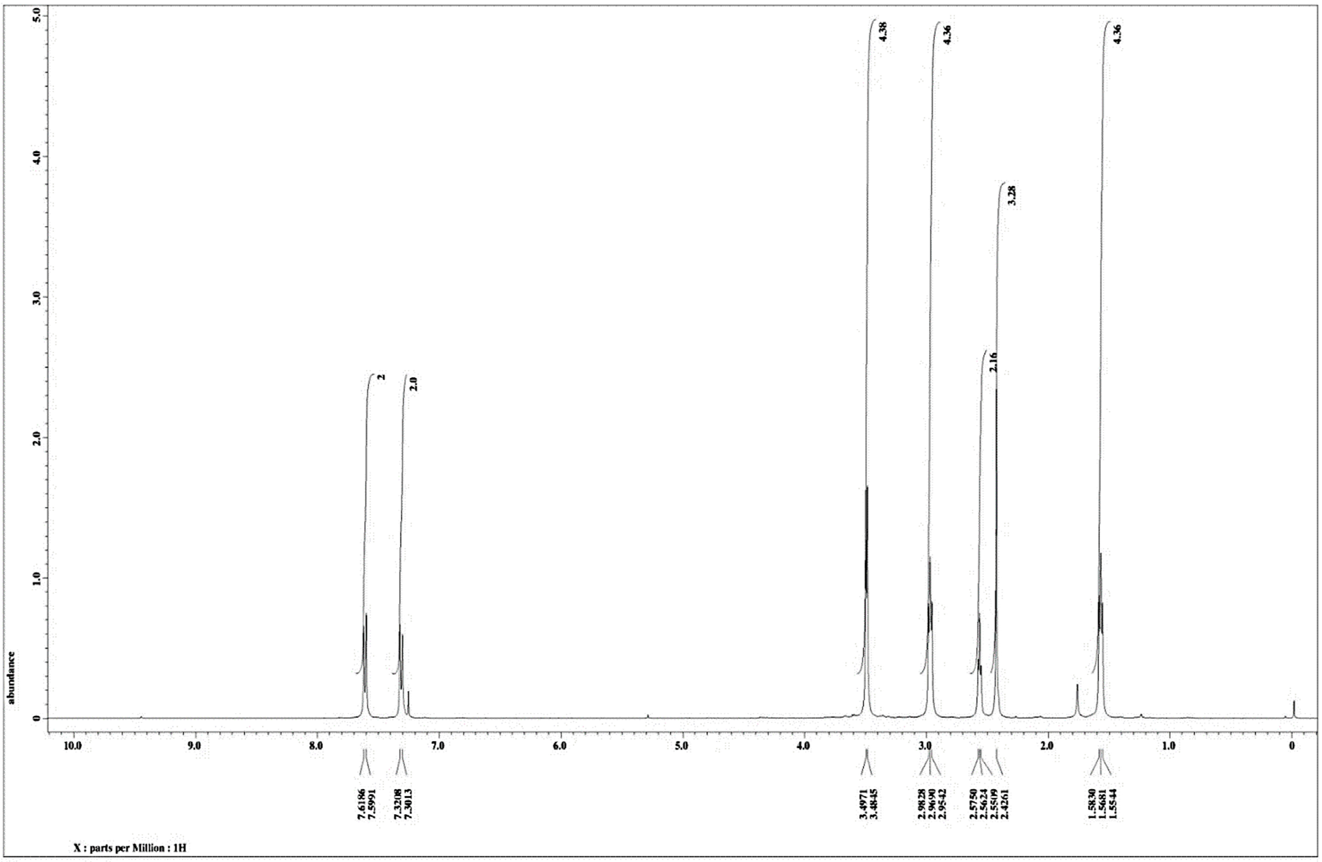

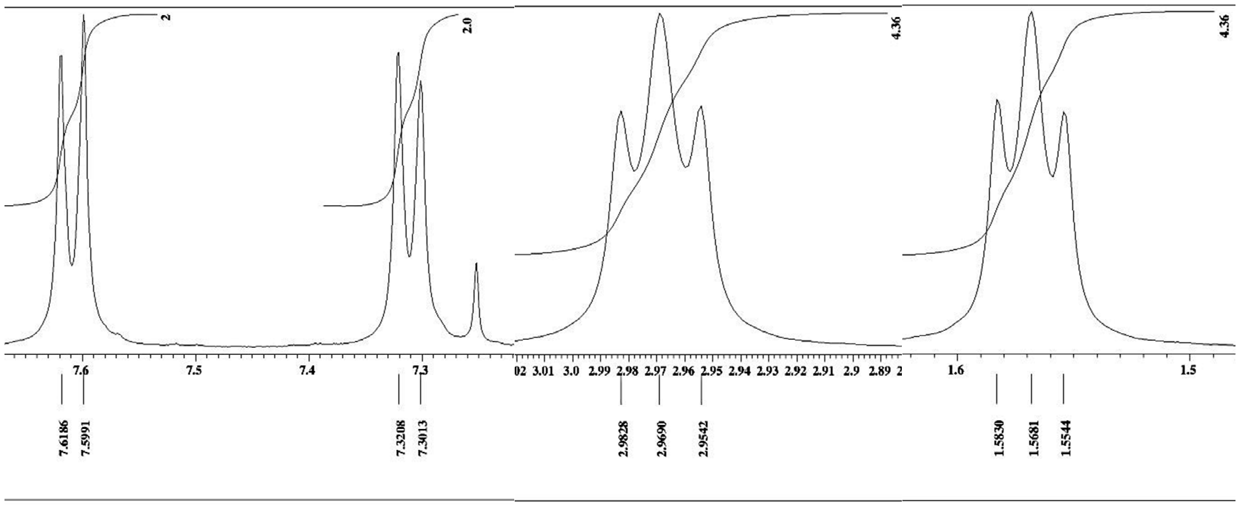

^{13}C-NMR (100 MHz) of [1-(4-Methylbenzene-1-sulfonyl)]piperidine-4,4-diyl]dimethanol (**4**) in $CDCl_3$

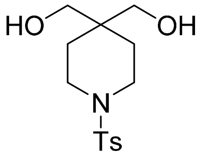

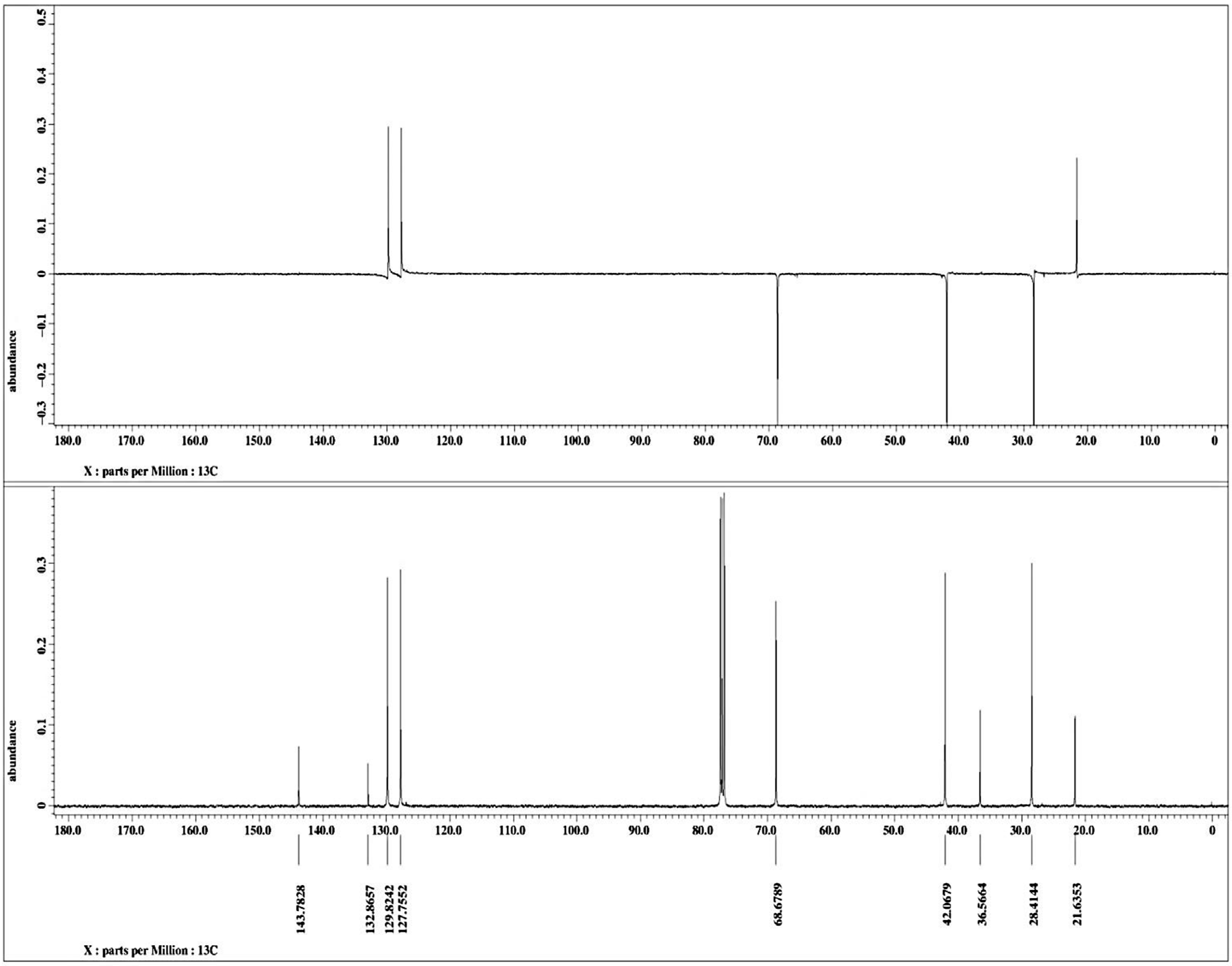

^{1}H-NMR (400 MHz) of 7-(4-Methylbenzene-1-sulfonyl)-2-oxa-7-azaspiro[3.5]nonane (**5**) in $CDCl_3$

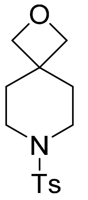

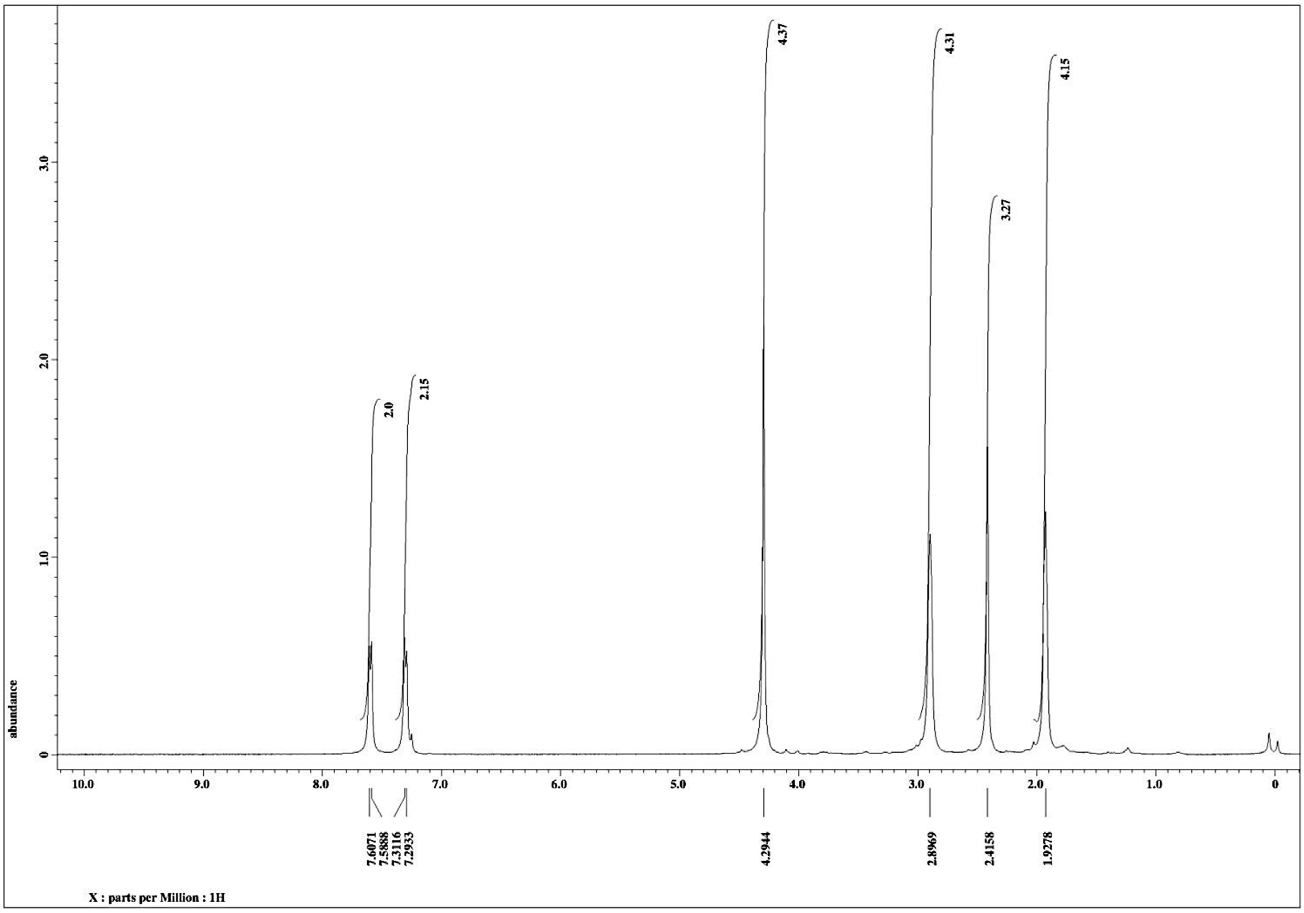

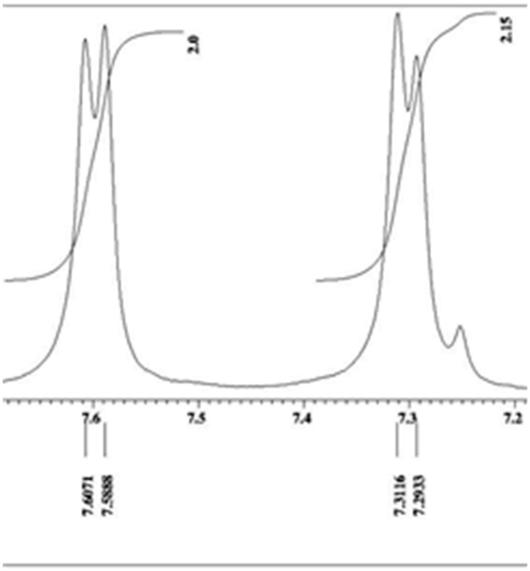

^{13}C-NMR (100 MHz) of 7-(4-Methylbenzene-1-sulfonyl)-2-oxa-7-azaspiro[3.5]nonane (**5**) in $CDCl_3$

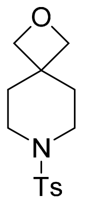

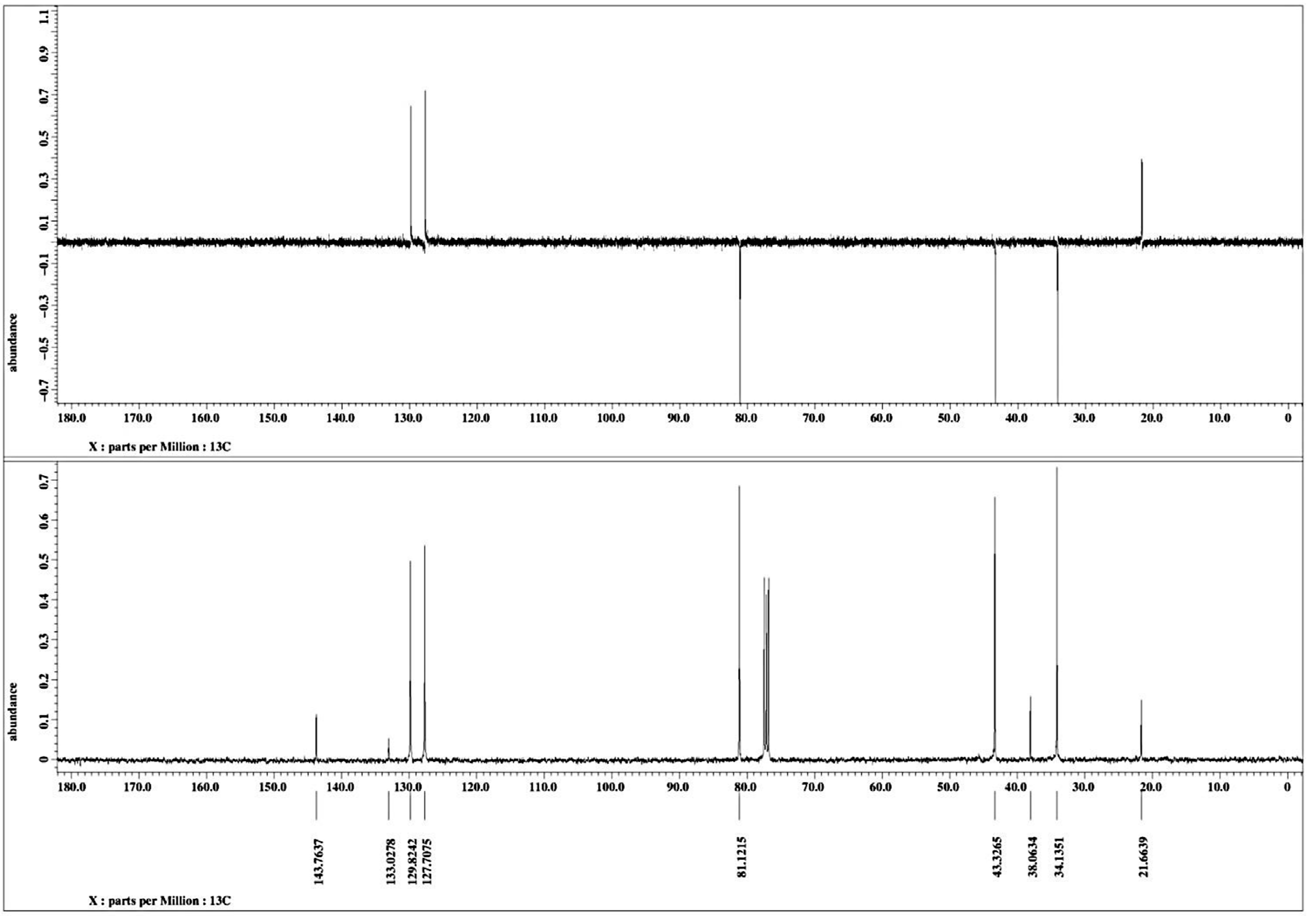

^{1}H-NMR (400 MHz) of Bis(2-oxa-7-azaspiro[3.5]nonan-7-ium) Ethanedioate (oxalate salt of **1b**) in D_2O

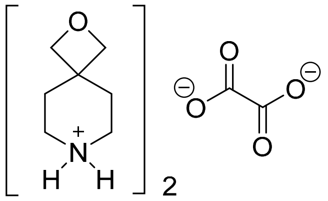

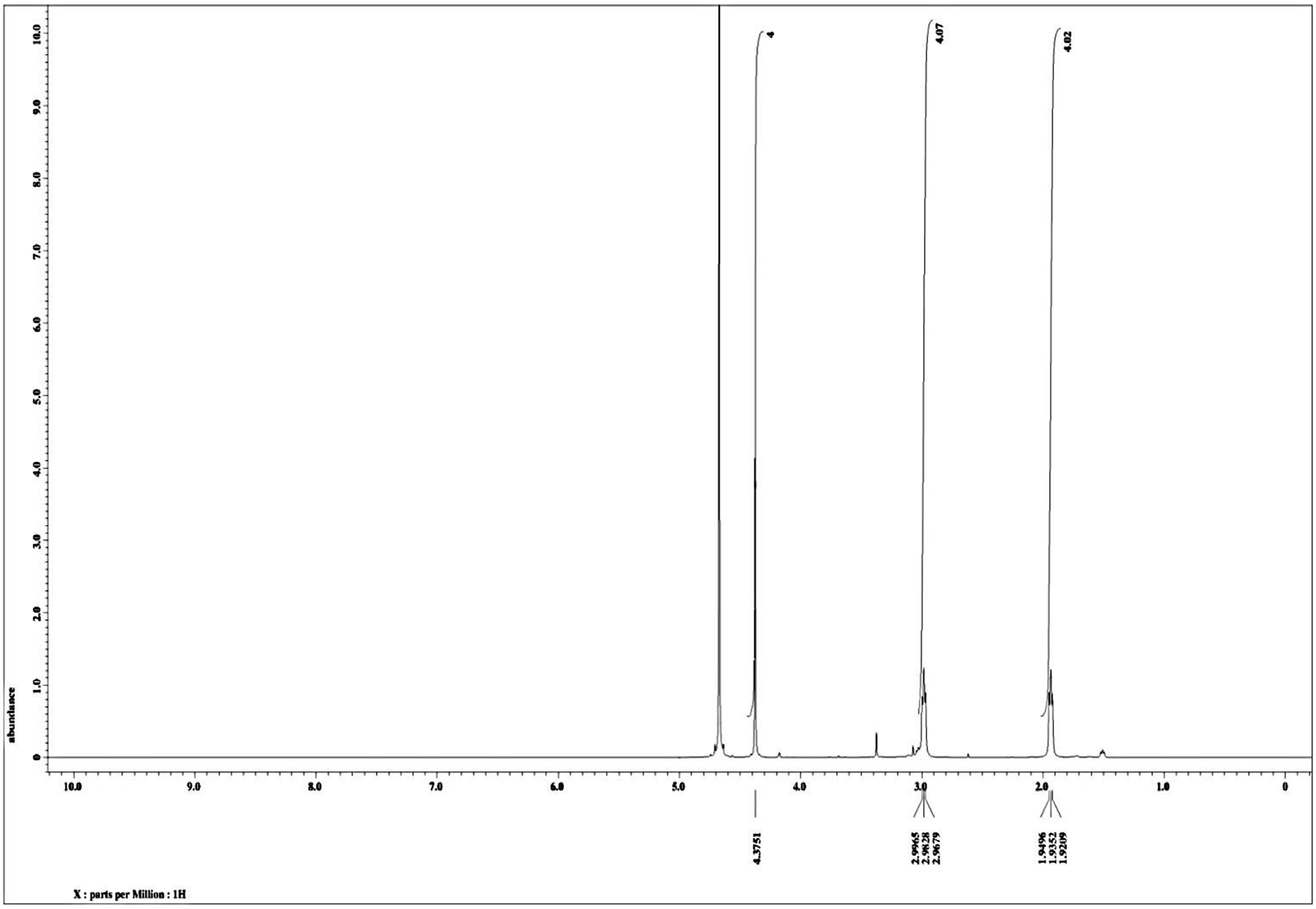

^{13}C-NMR (100 MHz) of Bis(2-oxa-7-azaspiro[3.5]nonan-7-ium) Ethanedioate (oxalate Salt of **1b**) in D_2O

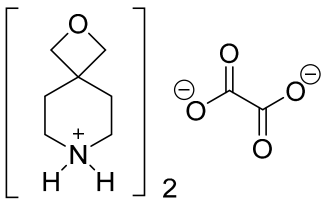

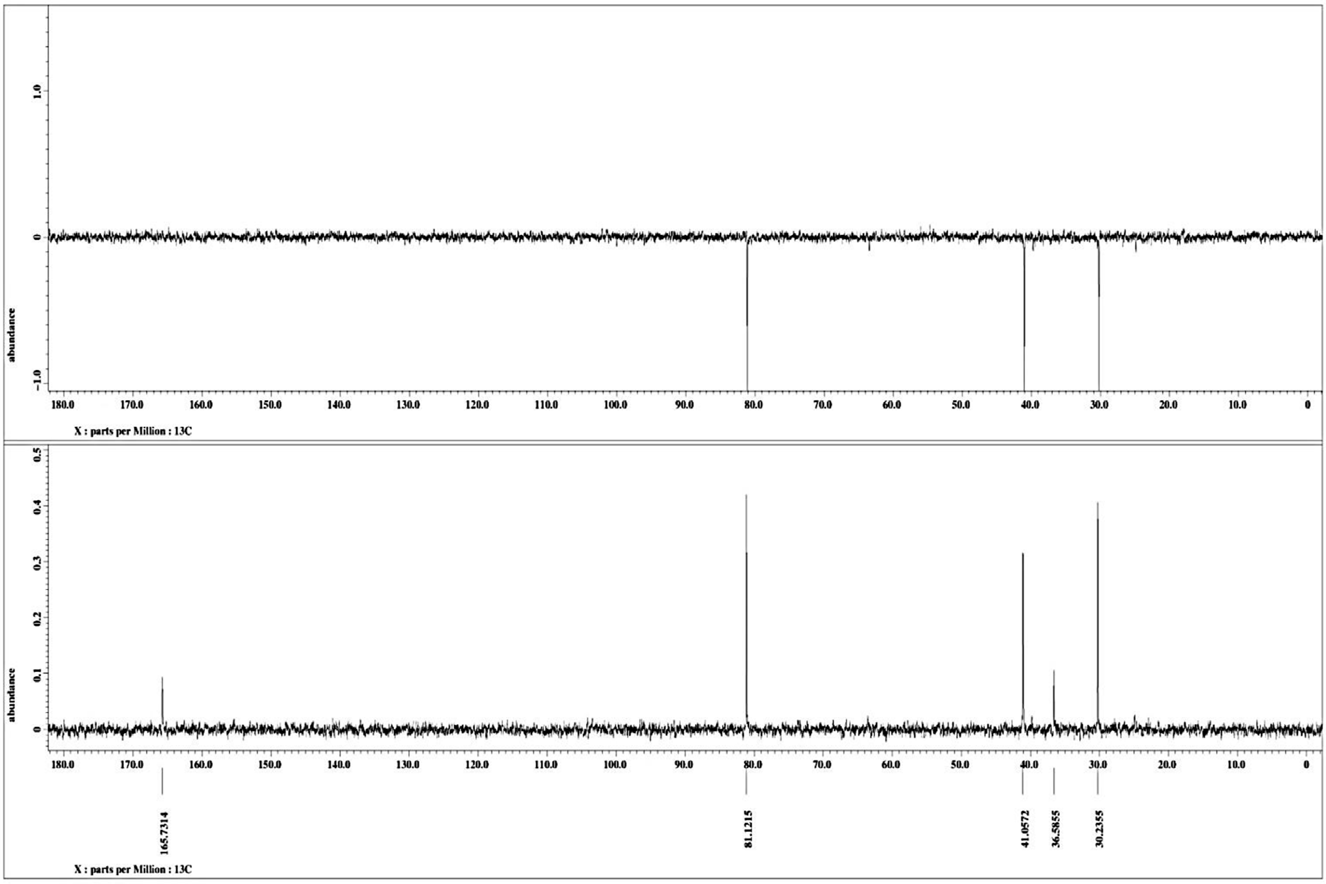

^{1}H-NMR (400 MHz) of 6-(4-Bromo-2-nitrophenyl)-2-oxa-6-azaspiro[3.3]heptane (**6a**) in $CDCl_3$

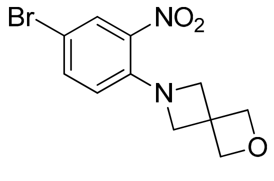

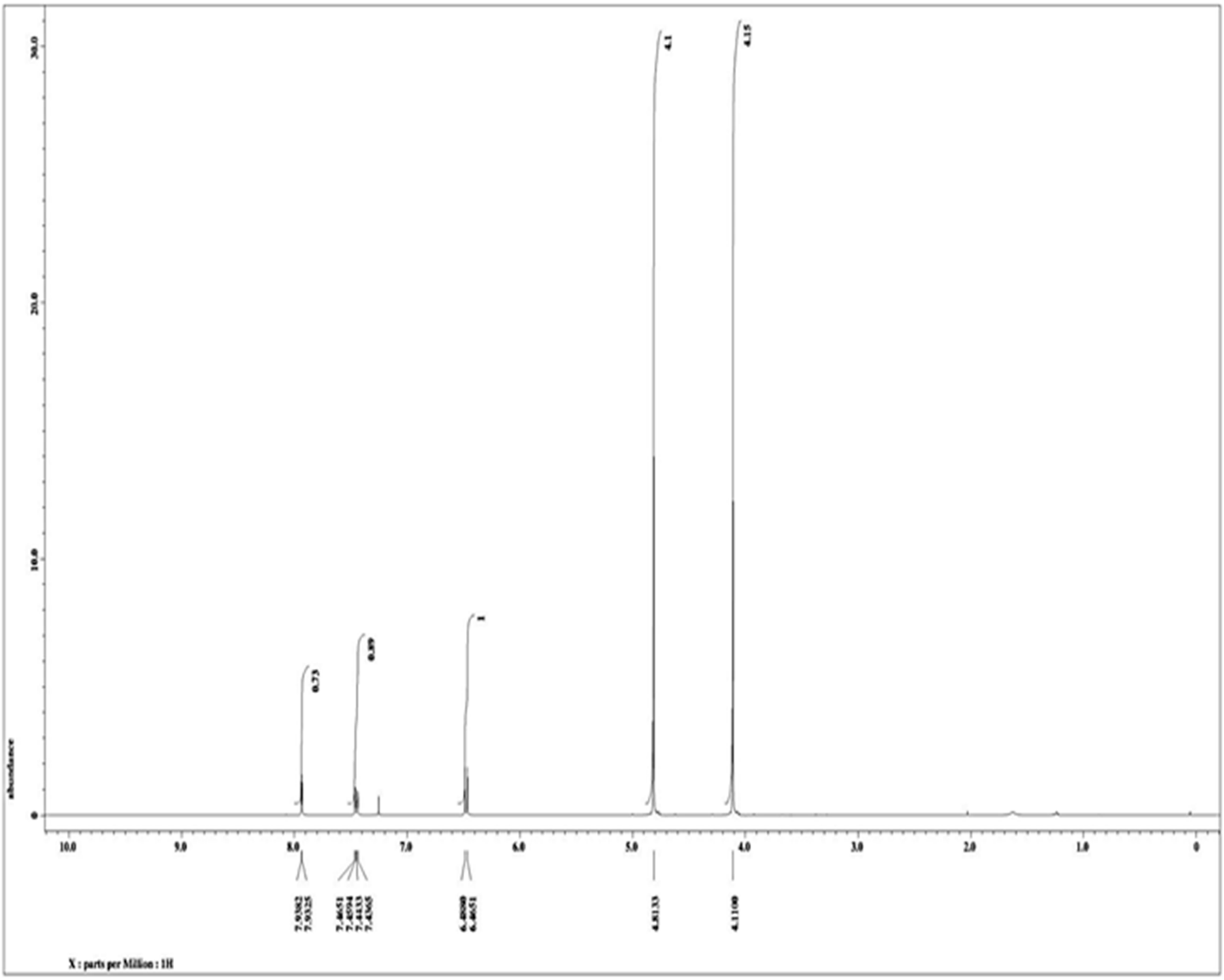

^{13}C-NMR (100 MHz) of 6-(4-Bromo-2-nitrophenyl)-2-oxa-6-azaspiro[3.3]heptane (**6a**) in $CDCl_3$

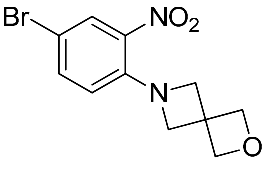

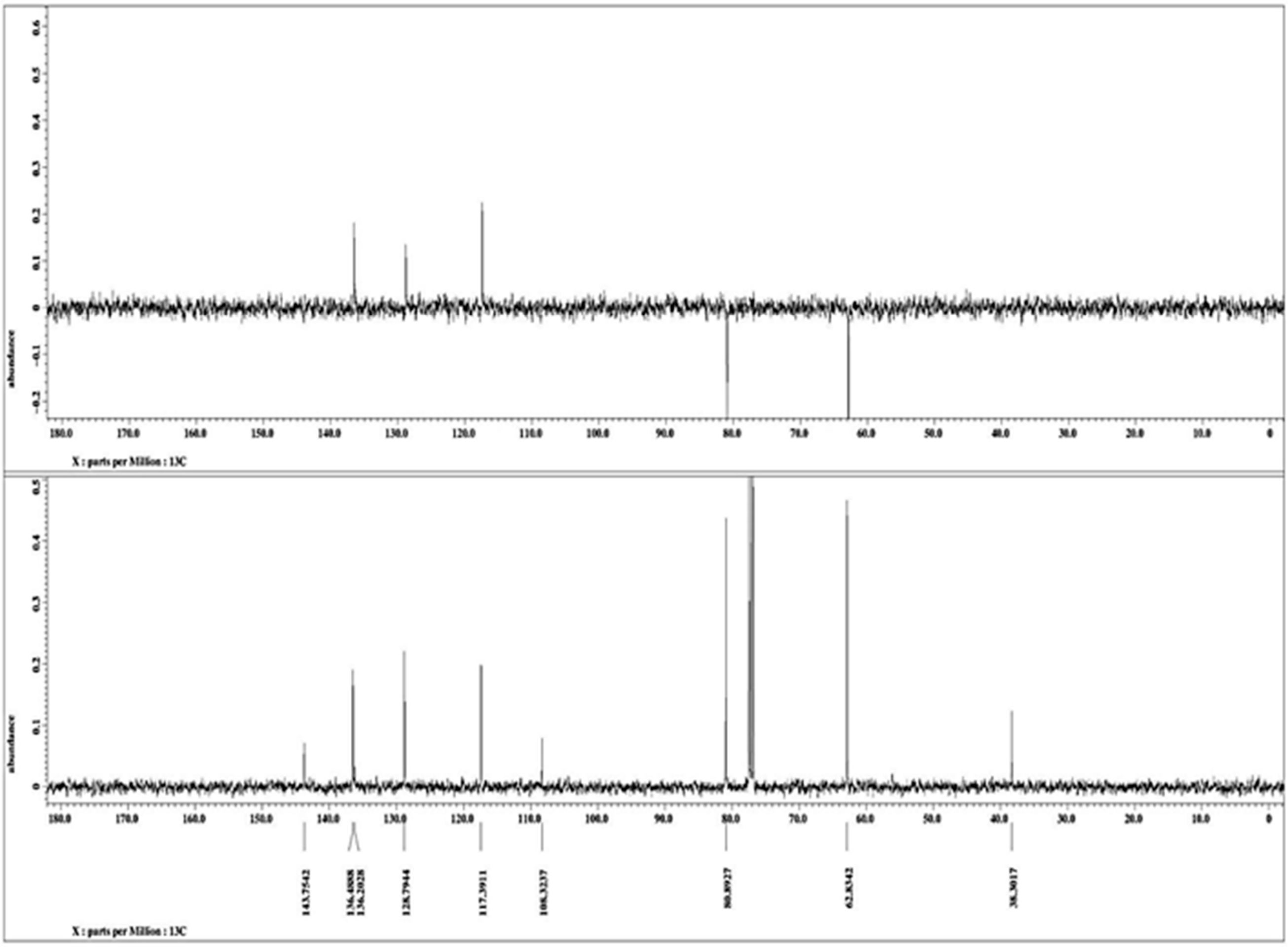

^{1}H-NMR (400 MHz) of 7-(4-Bromo-2-nitrophenyl)-2-oxa-7-azaspiro[3.5]nonane (**6b**) in $CDCl_3$

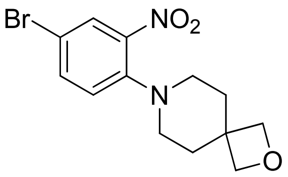

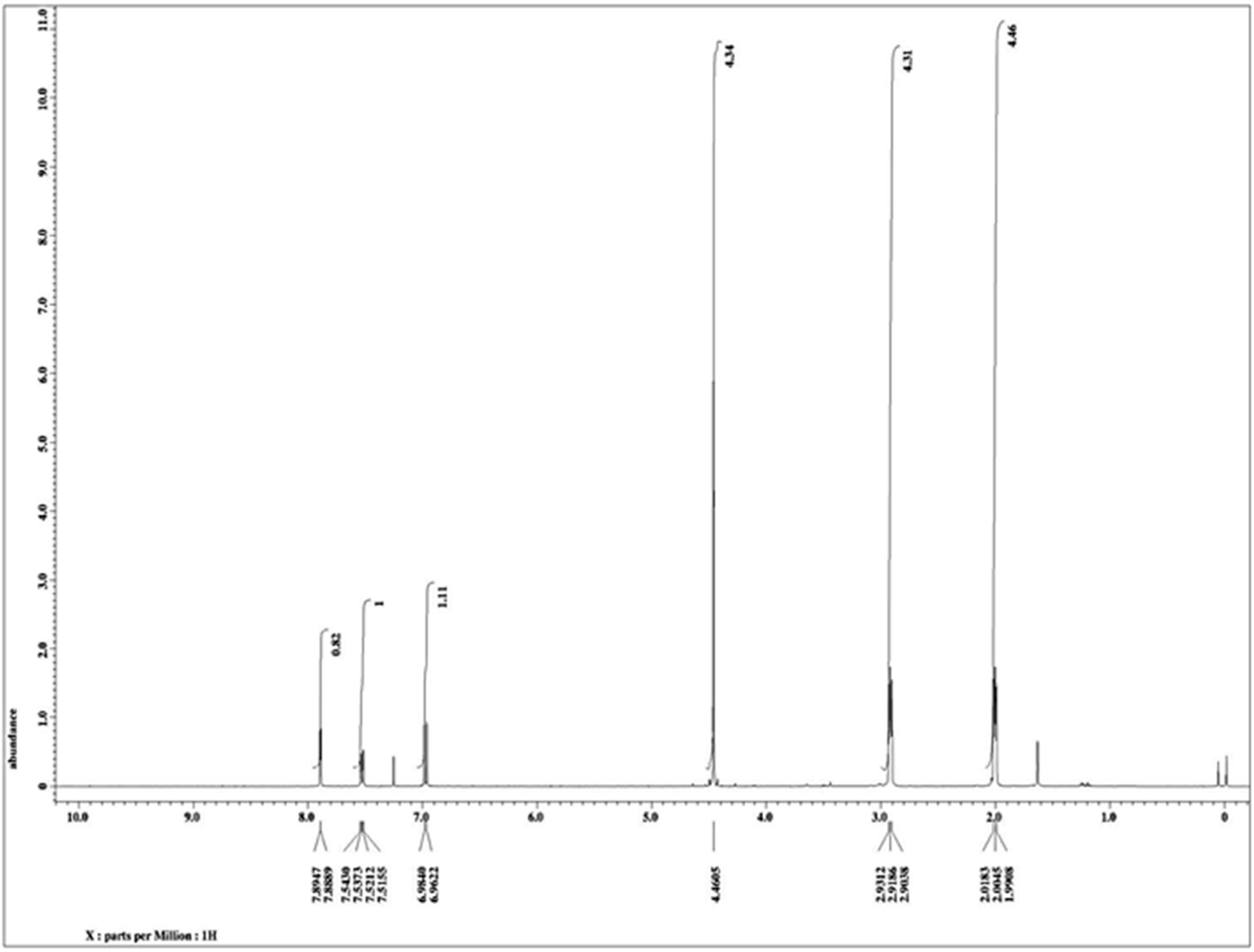

^{13}C-NMR (100 MHz) of 7-(4-Bromo-2-nitrophenyl)-2-oxa-7-azaspiro[3.5]nonane (**6b**) in $CDCl_3$

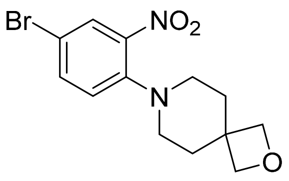

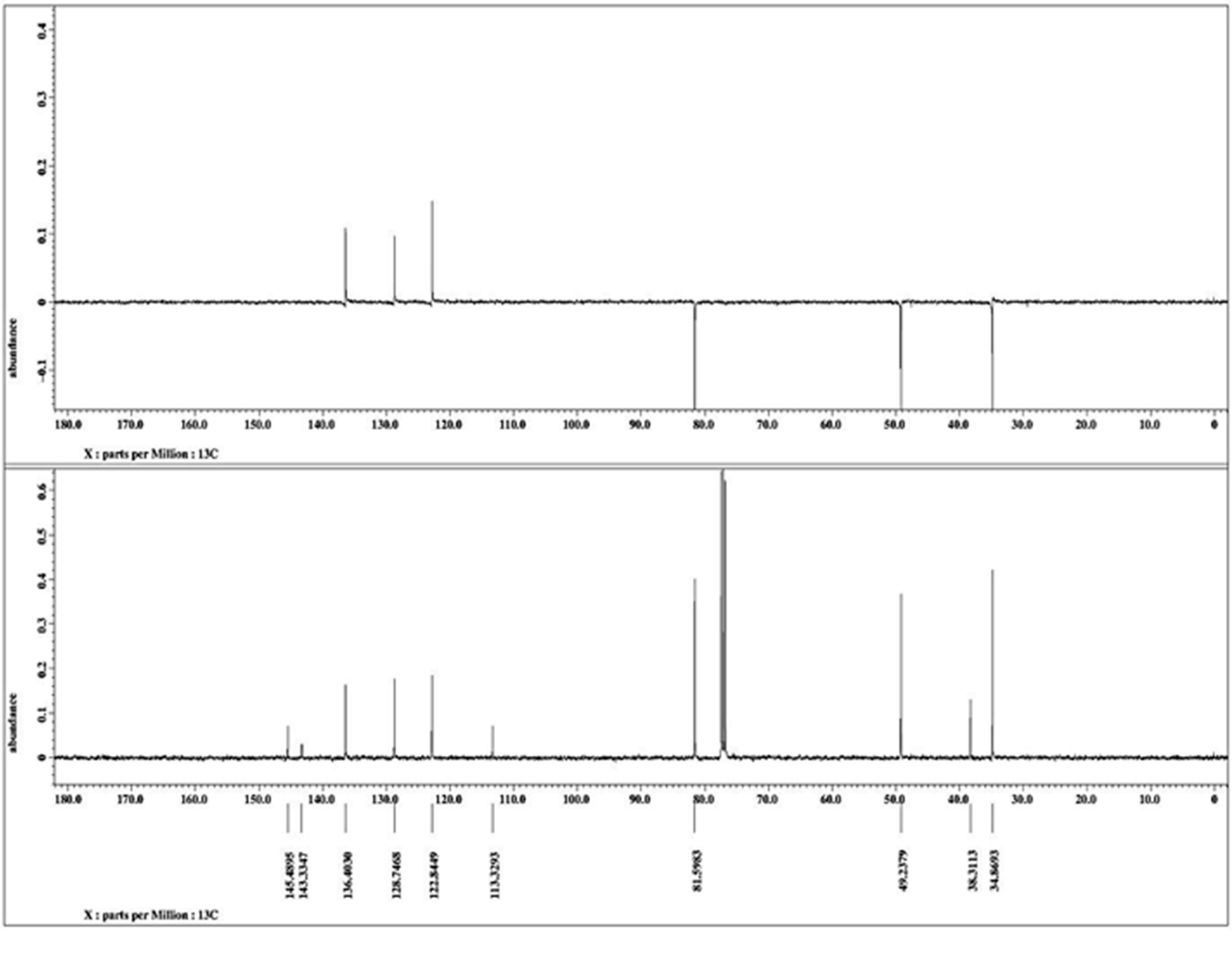

^{1}H-NMR (400 MHz) of 5-Bromo-2-(2-oxa-6-azaspiro[3.3]heptan-6-yl)aniline (**7a**) in $CDCl_3$

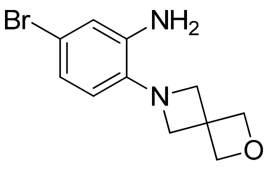

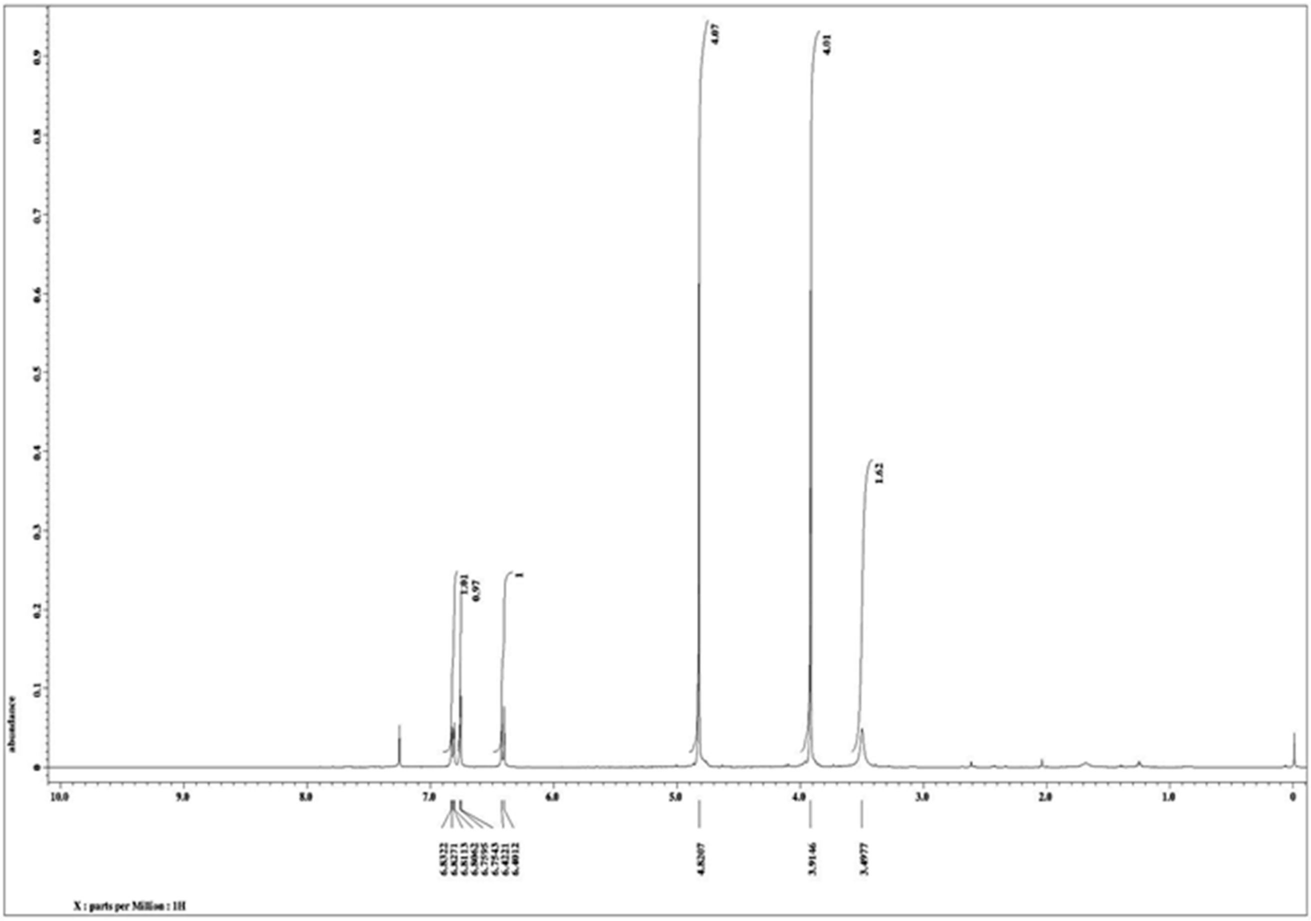

^{13}C-NMR (100 MHz) of 5-Bromo-2-(2-oxa-6-azaspiro[3.3]heptan-6-yl)aniline (**7a**) in $CDCl_3$

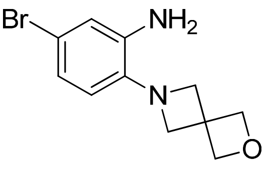

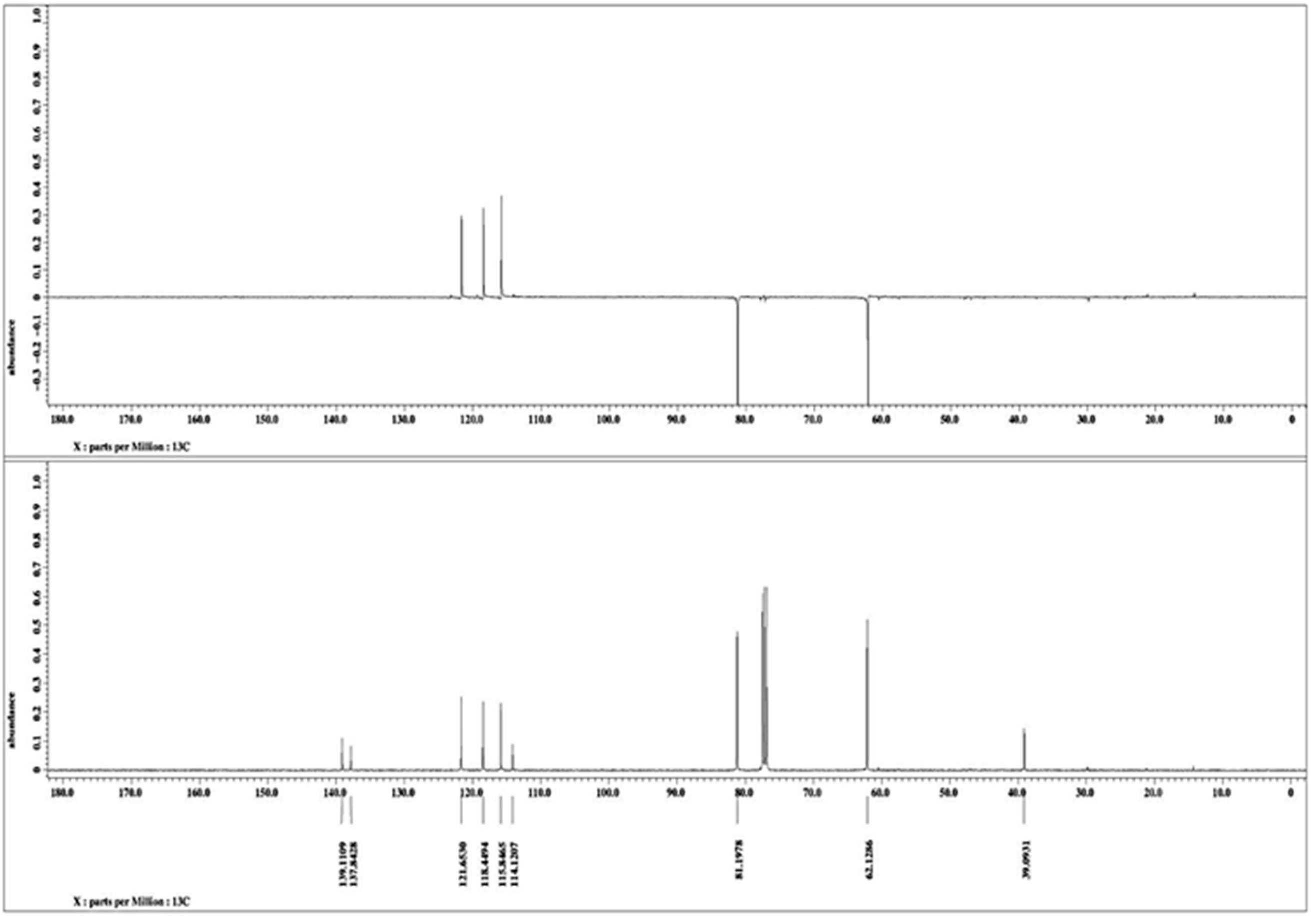

^{1}H-NMR (400 MHz) of 5-Bromo-2-(2-oxa-7-azaspiro[3.5]nonan-7-yl)aniline (**7b**) in $CDCl_3$

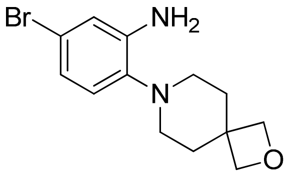

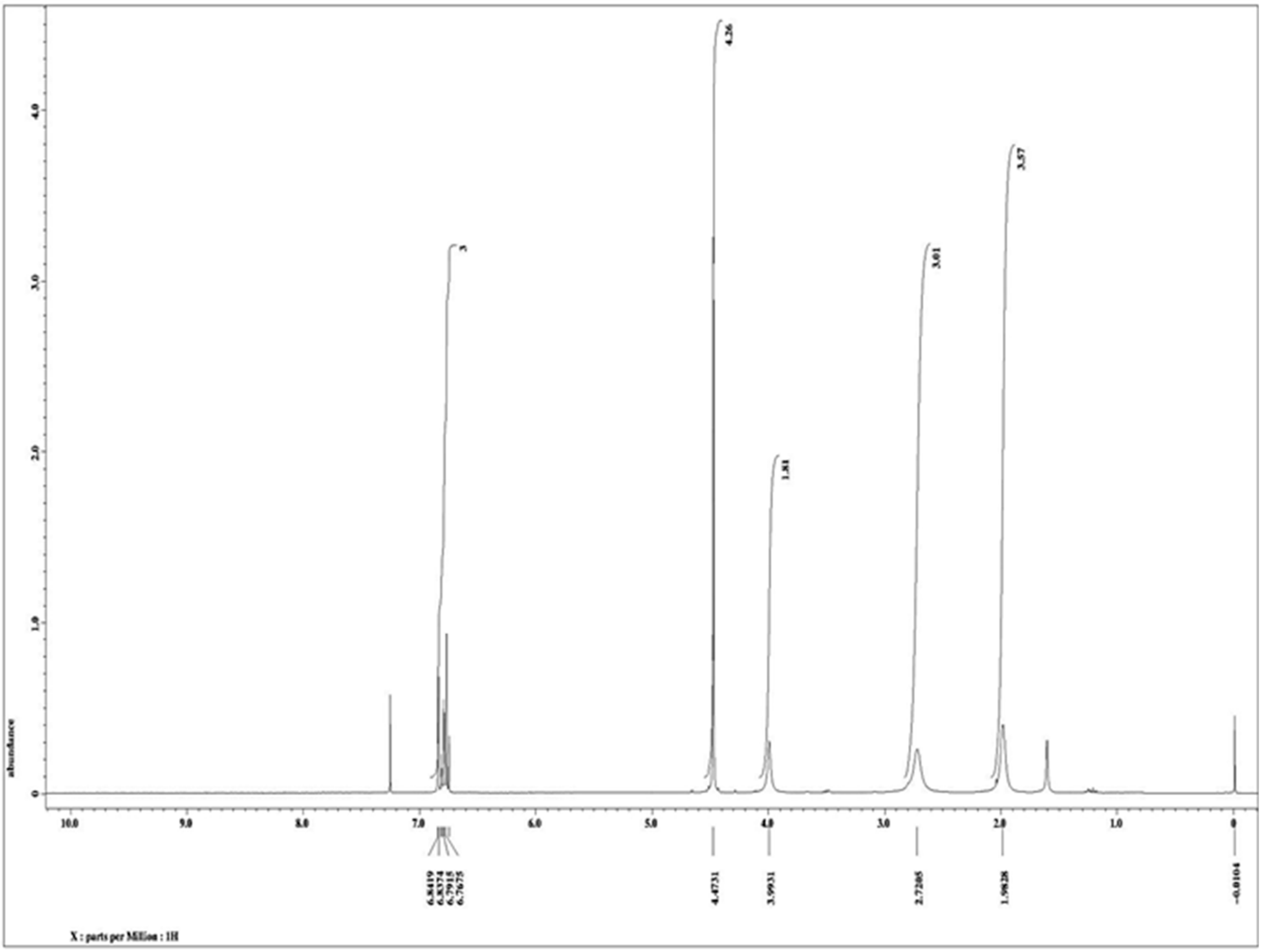

^{13}C-NMR (100 MHz) of 5-Bromo-2-(2-oxa-7-azaspiro[3.5]nonan-7-yl)aniline (**7b**) in $CDCl_3$

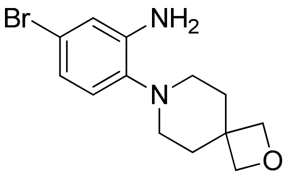

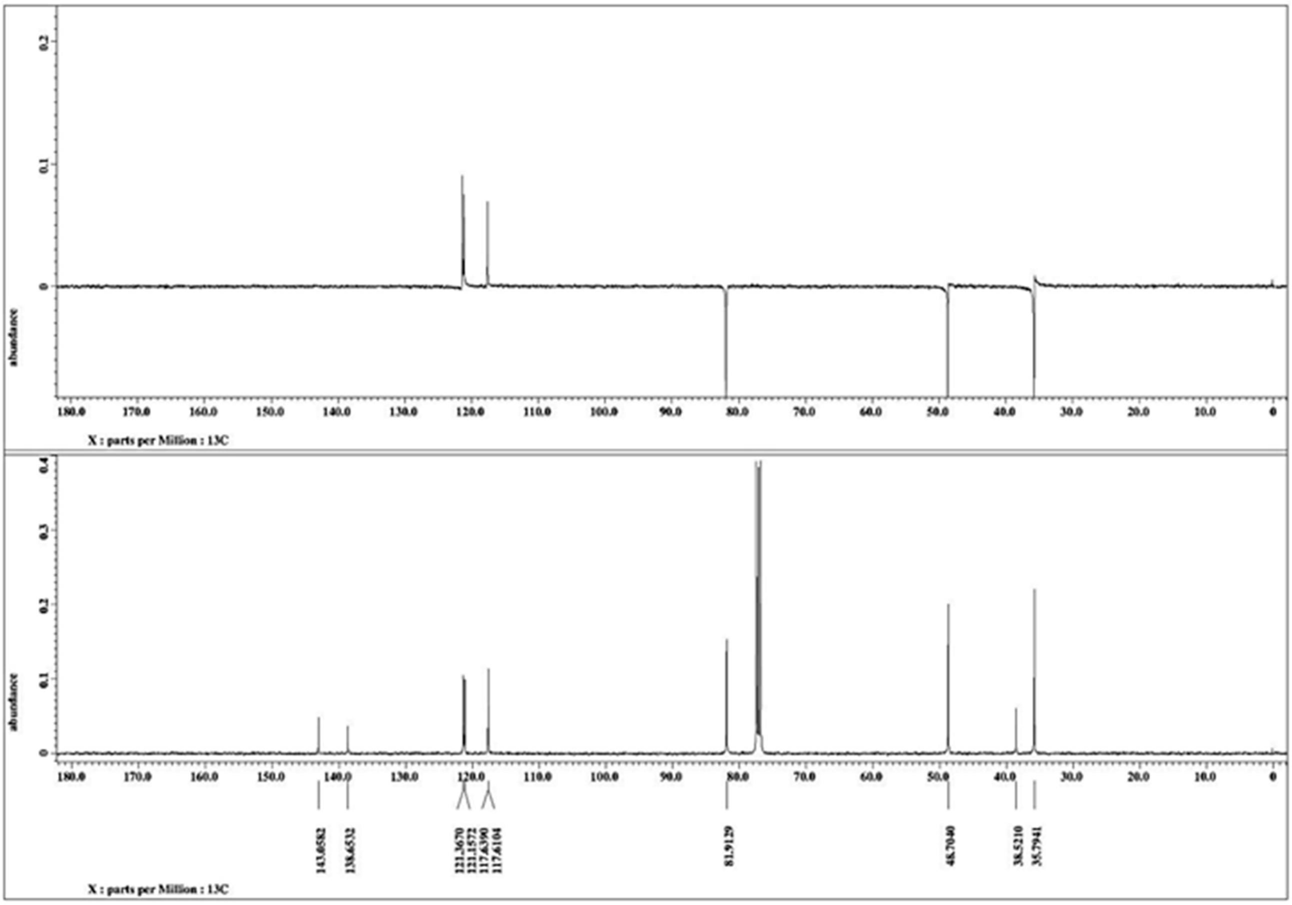

^{1}H-NMR (400 MHz) of *N*-(2-Acetylamido-4-bromophenyl)-*N*-{[3-(chloromethyl)oxetan-3-yl]methyl} acetamide (**9a**) in $CDCl_3$

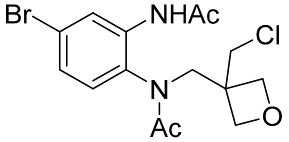

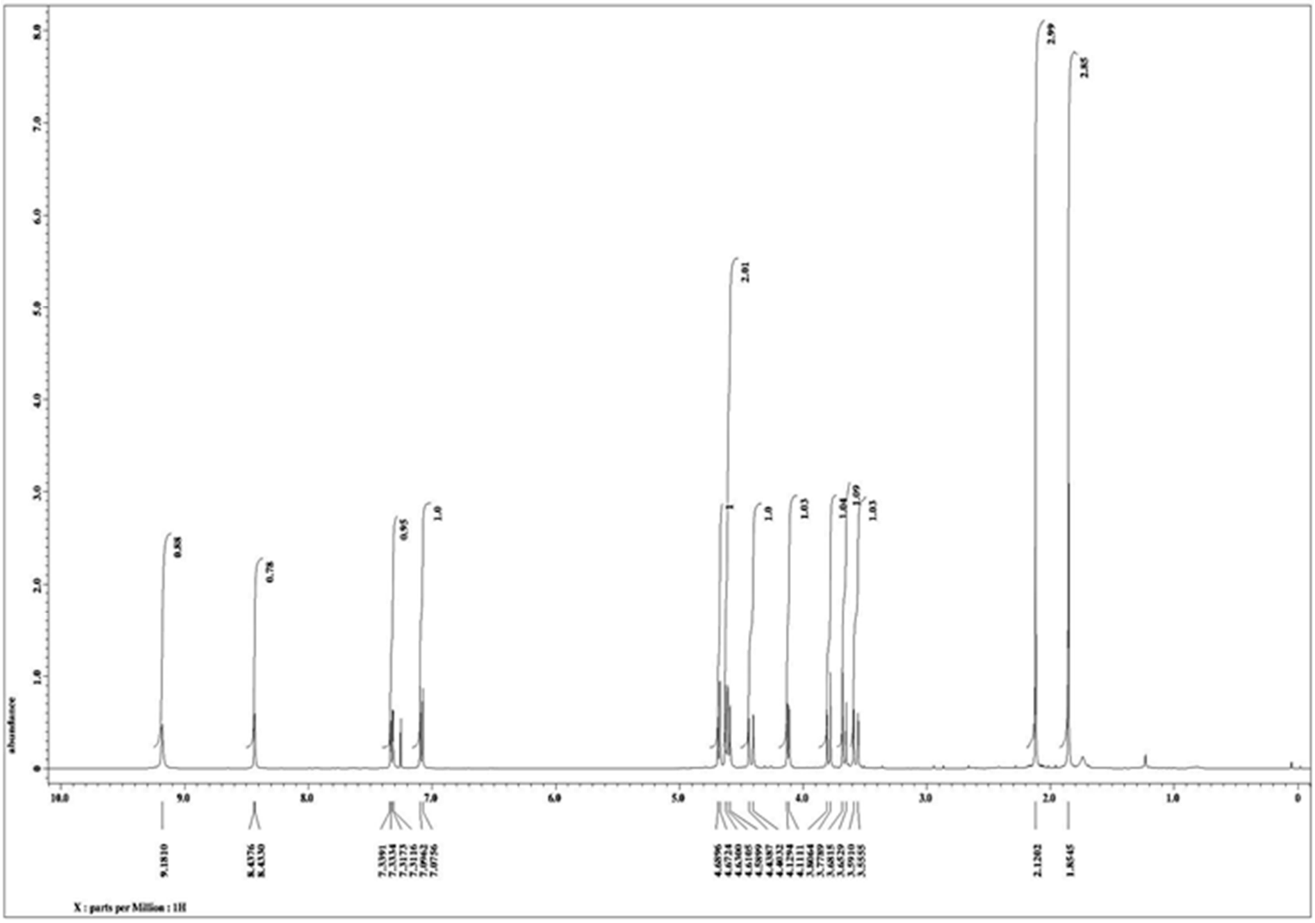

^{13}C-NMR (100 MHz) of *N*-(2-Acetylamido-4-bromophenyl)-*N*-{[3-(chloromethyl)oxetan-3-yl]methyl} acetamide (**9a**) in $CDCl_3$

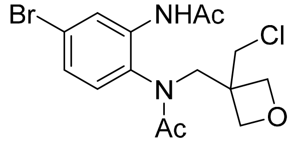

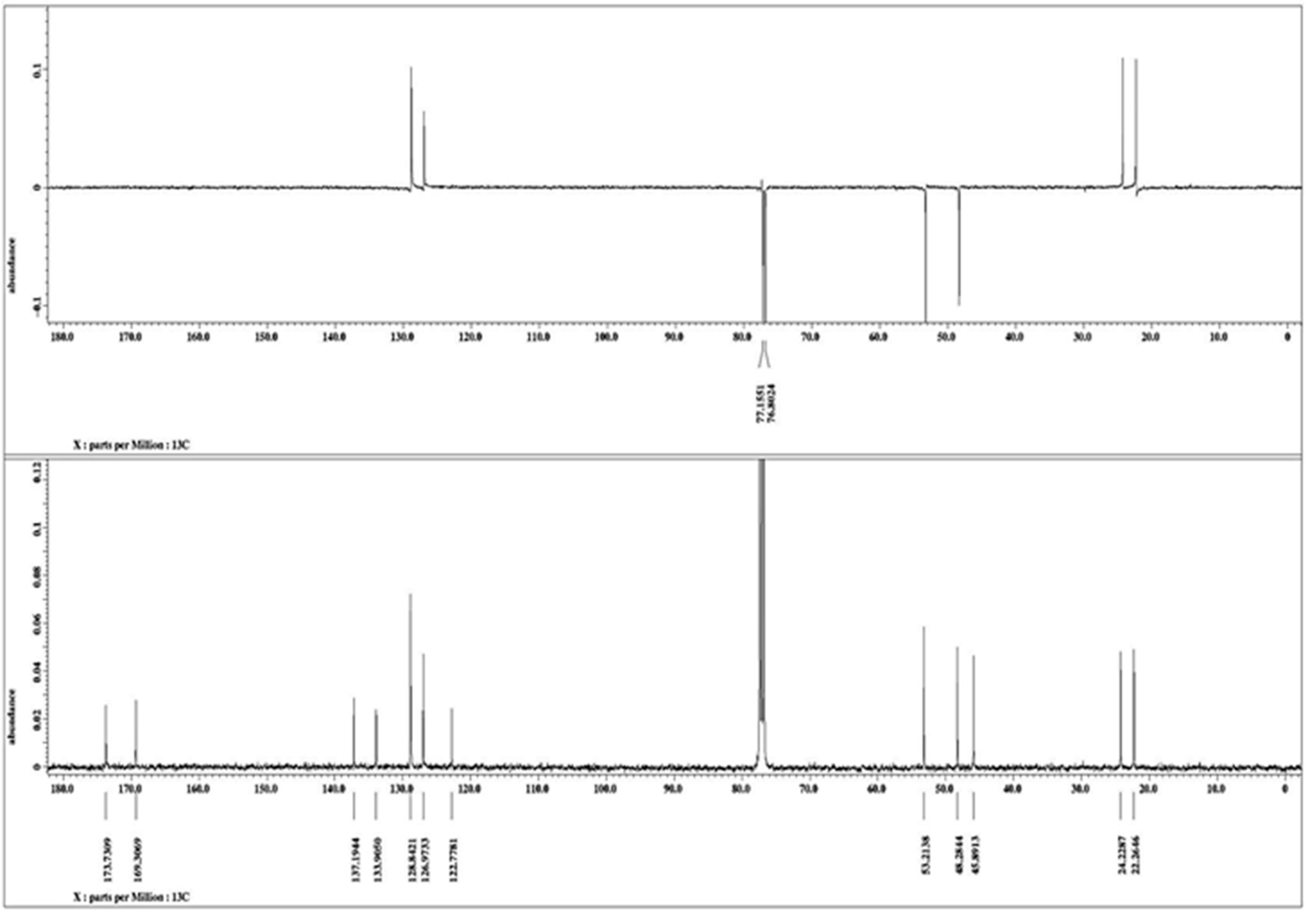

^{1}H-NMR (400 MHz) of *N*-[5-Bromo-2-(2-oxa-6-azaspiro[3.3]heptan-6-yl)phenyl]acetamide (**8a**) in $(CD_3)_2SO$

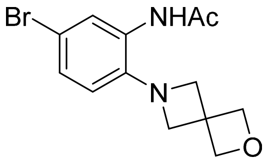

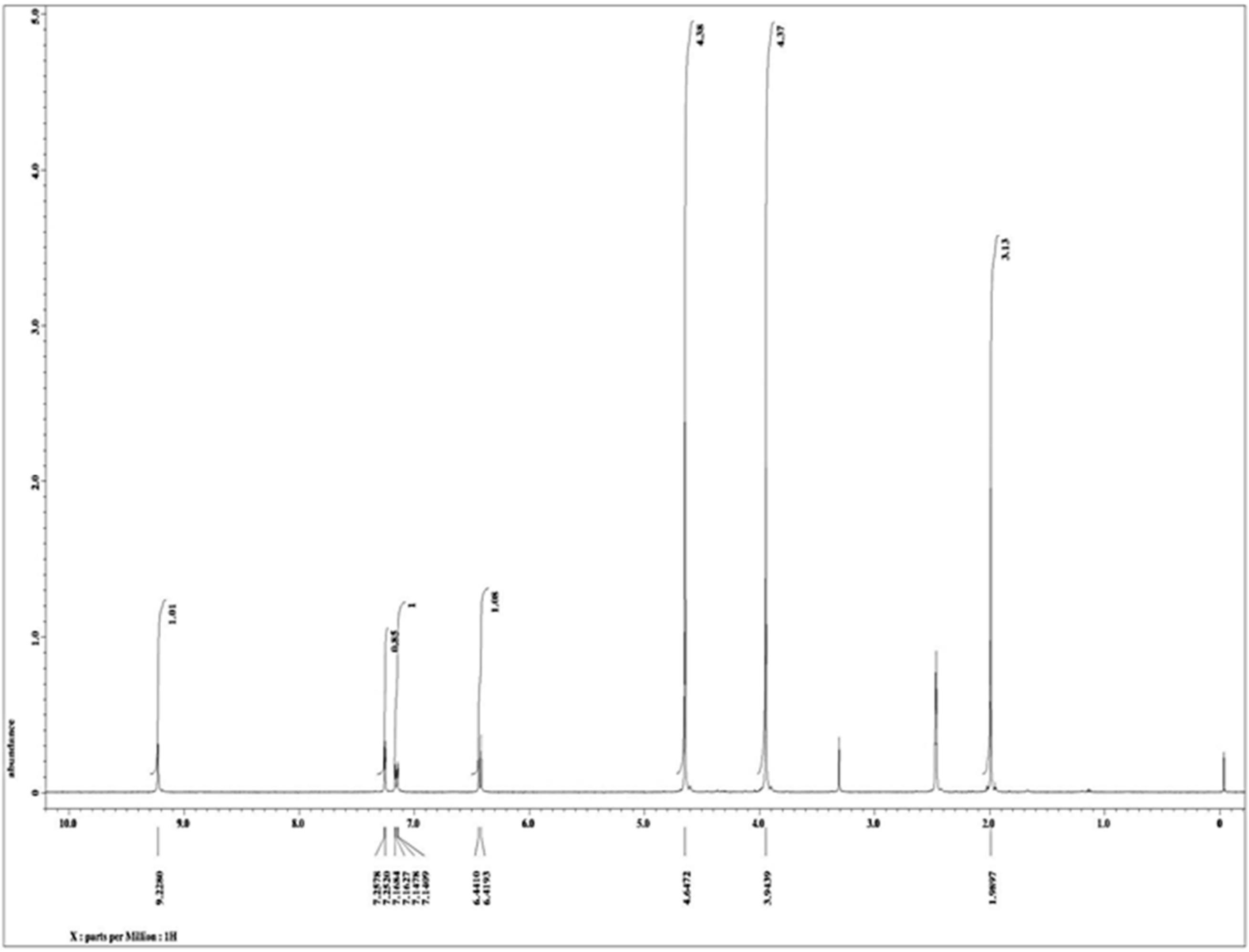

^{13}C-NMR (100 MHz) of *N*-[5-Bromo-2-(2-oxa-6-azaspiro[3.3]heptan-6-yl)phenyl]acetamide (**8a**) in $(CD_3)_2SO$

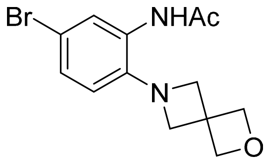

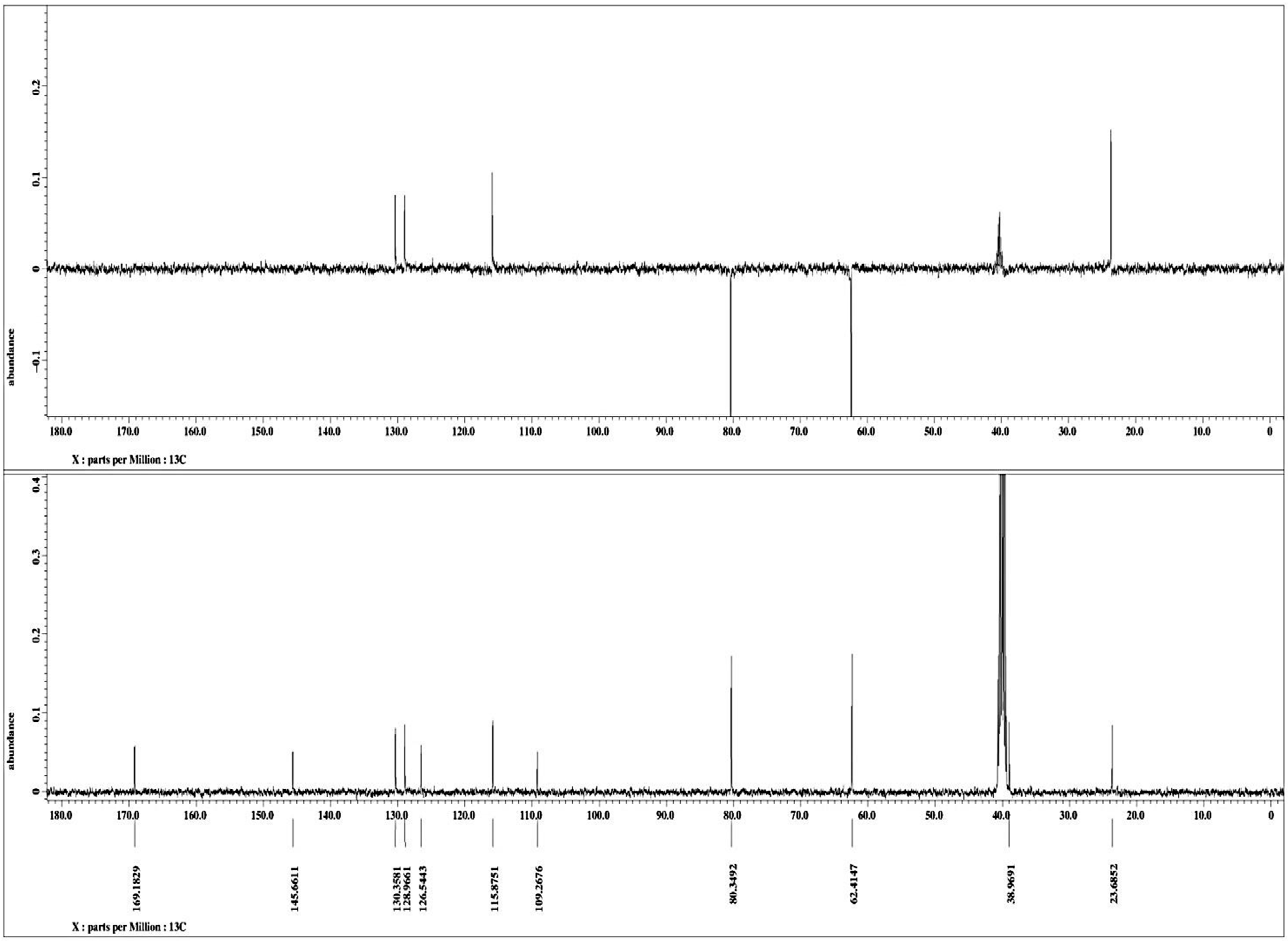

^{1}H-NMR (400 MHz) of *N*-Acetyl-*N*-[5-bromo-2-(2-oxa-6-azaspiro[3.3]heptan-6-yl)phenyl]acetamide (**10a**) in $CDCl_3$

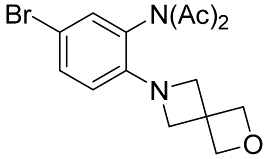

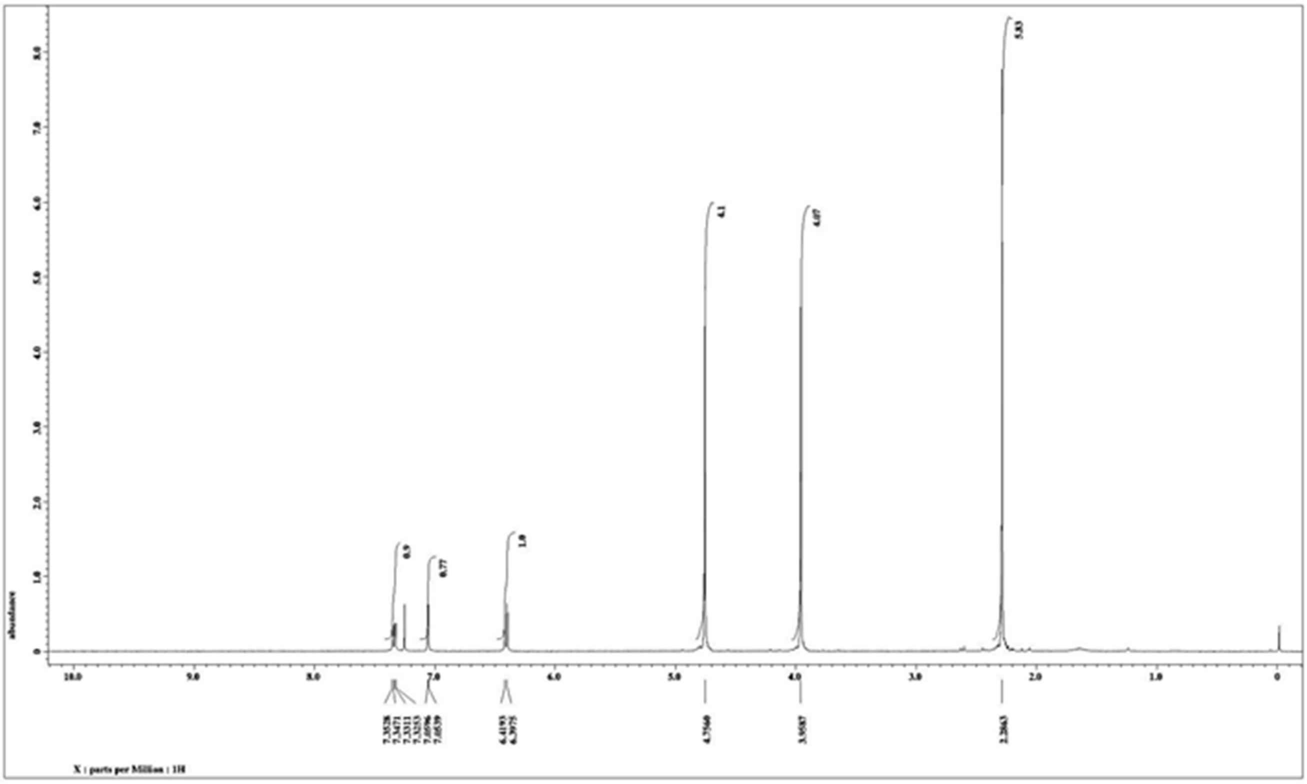

^{13}C-NMR (100 MHz) of *N*-Acetyl-*N*-[5-bromo-2-(2-oxa-6-azaspiro[3.3]heptan-6-yl)phenyl]acetamide (**10a**) in $CDCl_3$

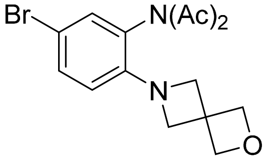

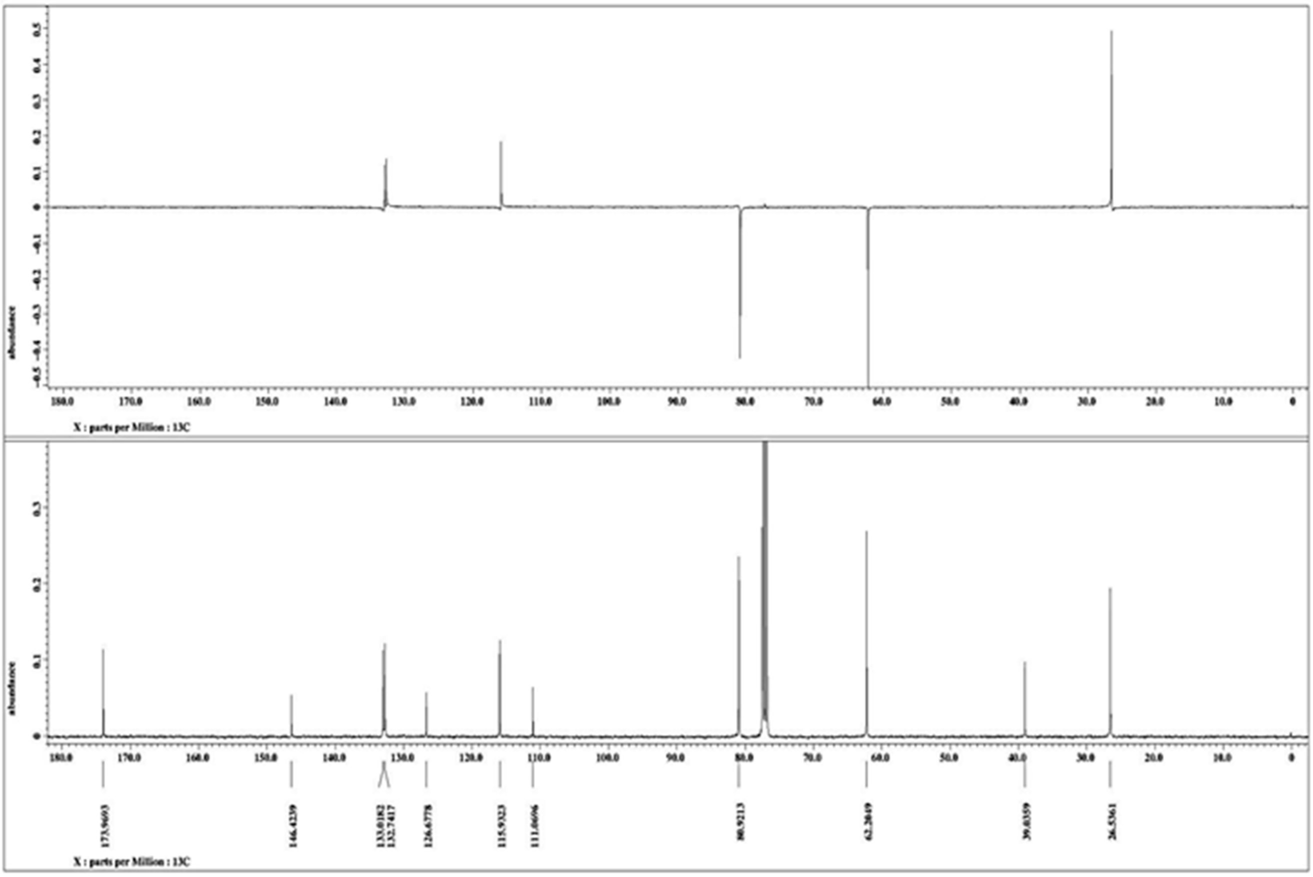

^{1}H-NMR (400 MHz) of *N*-[5-Bromo-2-(2-oxa-7-azaspiro[3.5]nonan-7-yl)phenyl]acetamide (**8b**) in $CDCl_3$

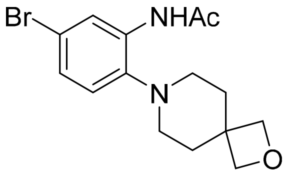

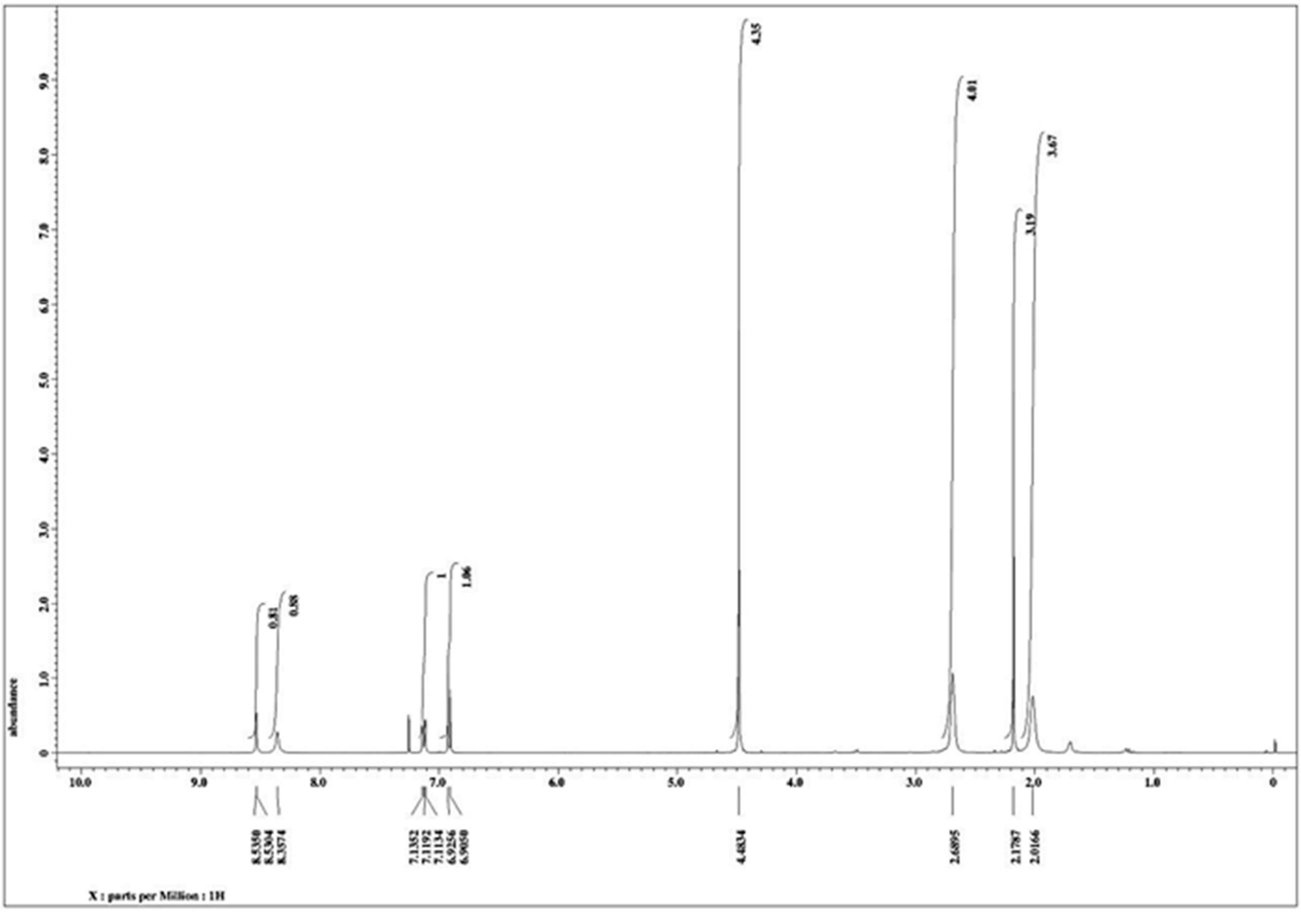

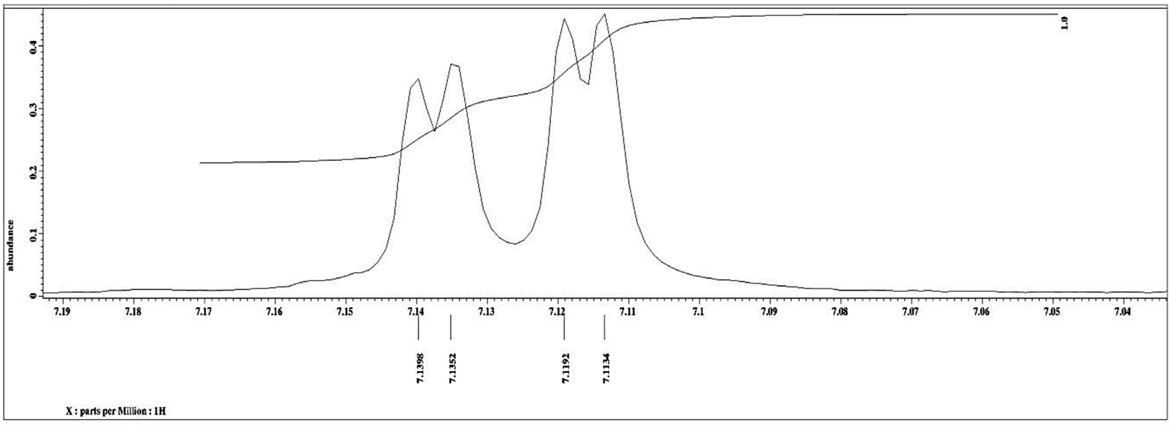

^{13}C-NMR (100 MHz) of *N*-[5-Bromo-2-(2-oxa-7-azaspiro[3.5]nonan-7-yl)phenyl]acetamide (**8b**) in $CDCl_3$

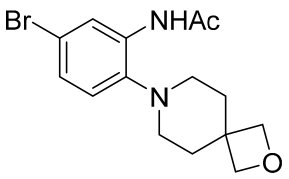

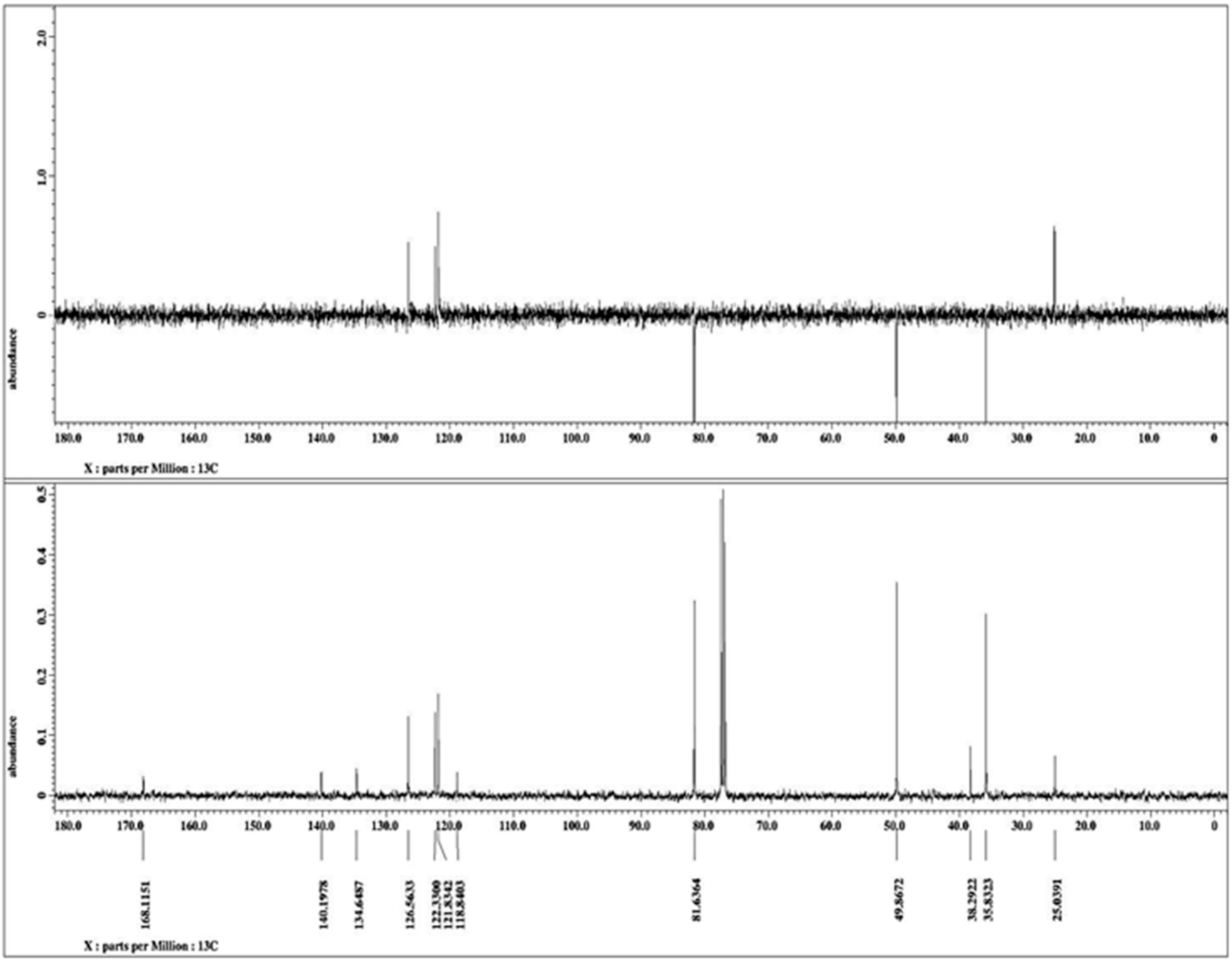

^{1}H-NMR (400 MHz) of 7′-Bromo-1′,2′-dihydro-4′*H*-spiro[oxetane-3,3′-pyrido[1,2-a]benzimidazole] (**2b**) in $CDCl_3$

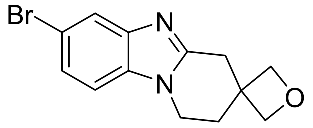

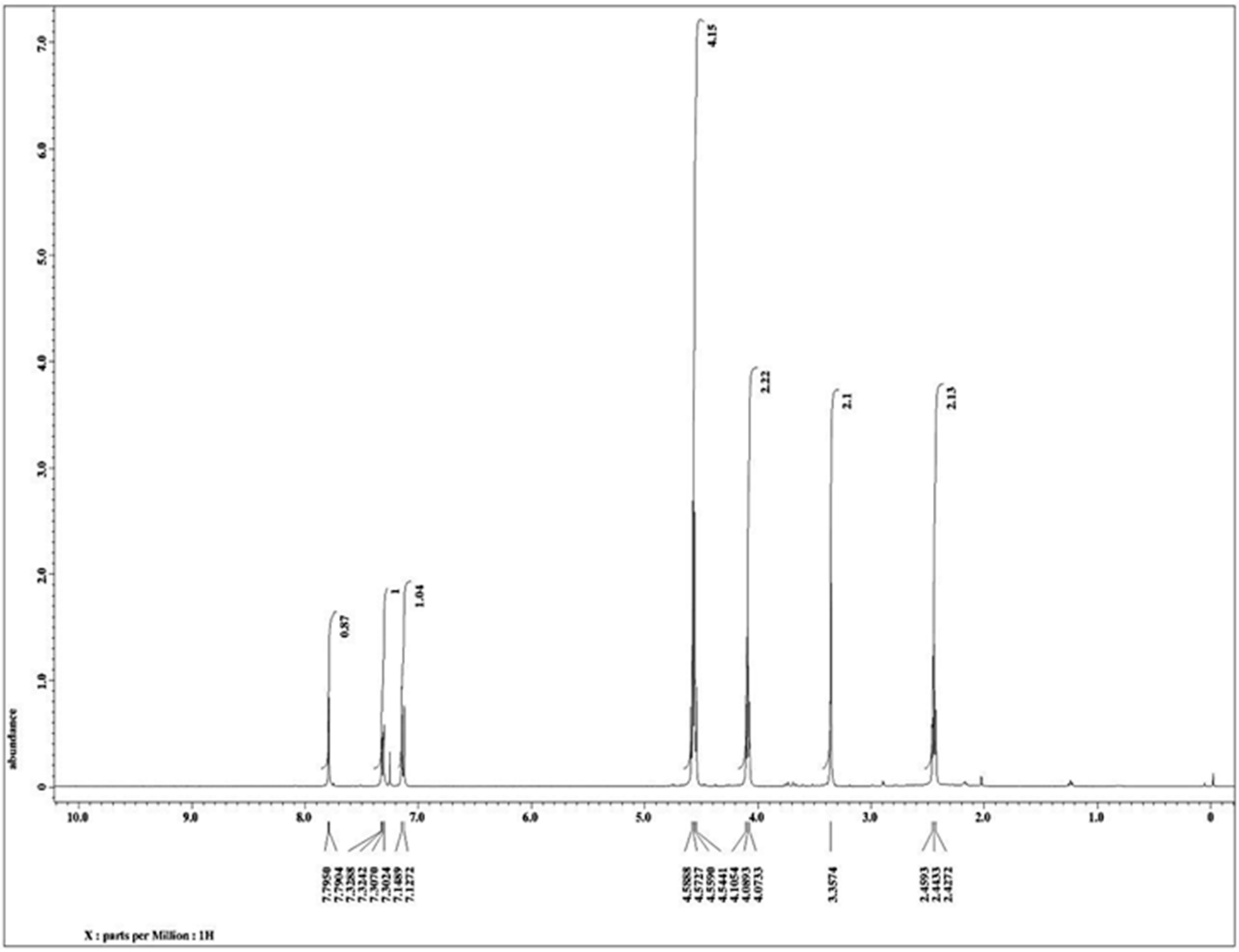

^{13}C-NMR (100 MHz) of 7′-Bromo-1′,2′-dihydro-4′*H*-spiro[oxetane-3,3′-pyrido[1,2-a]benzimidazole] (**2b**) in $CDCl_3$

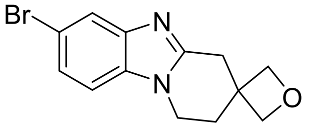

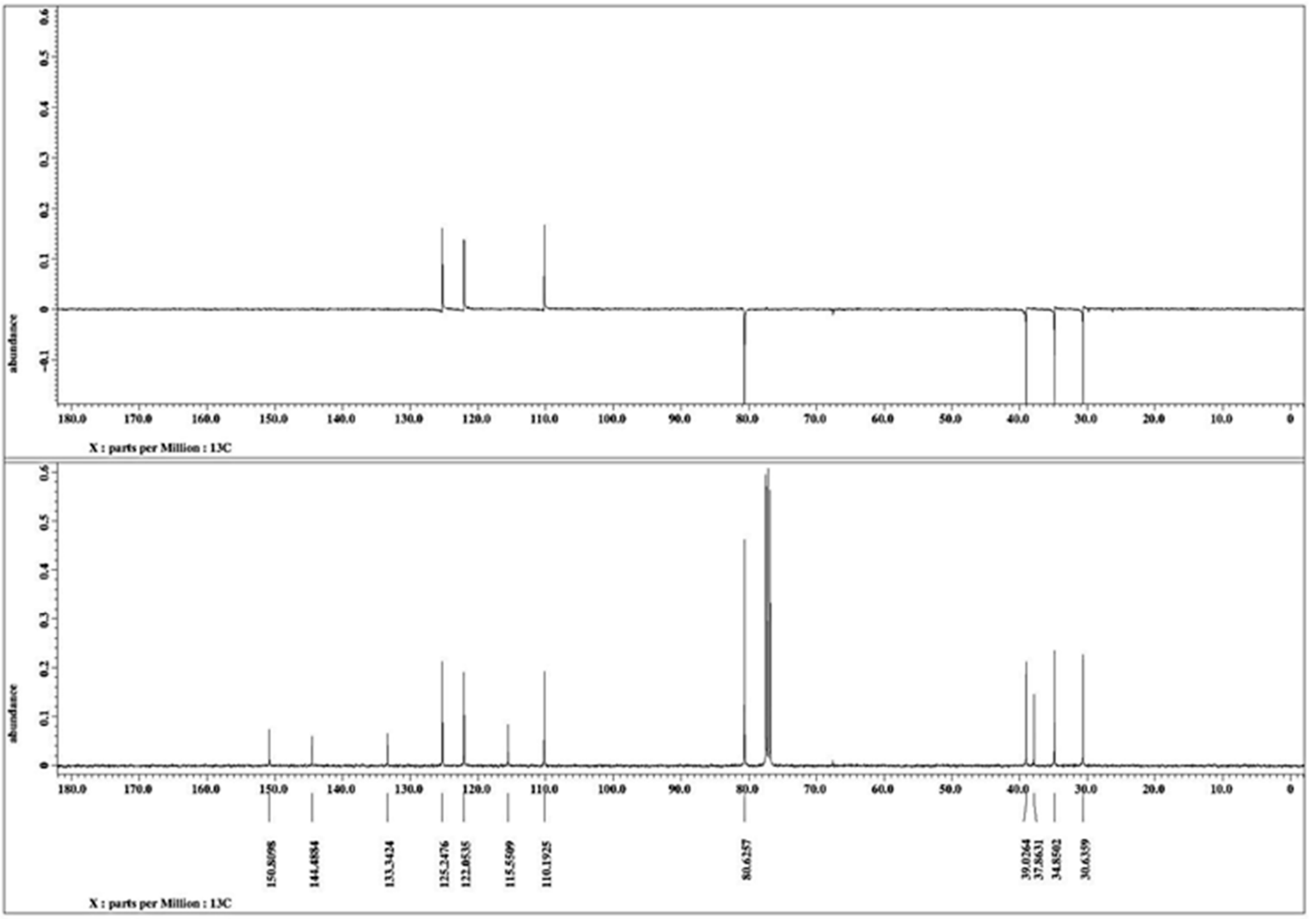

Supplement: Supplementary file 1 [file molecules-20-13864-s001.pdf]
